# Spectral Characterization of Bennu Analogs Using PASCALE: A New Experimental Set‐Up for Simulating the Near‐Surface Conditions of Airless Bodies

**DOI:** 10.1029/2020JE006624

**Published:** 2021-02-18

**Authors:** K. L. Donaldson Hanna, N. E. Bowles, T. J. Warren, V. E. Hamilton, D. L. Schrader, T. J. McCoy, J. Temple, A. Clack, S. Calcutt, D. S. Lauretta

**Affiliations:** ^1^ Department of Physics University of Central Florida Orlando FL USA; ^2^ Atmospheric Oceanic and Planetary Physics University of Oxford Oxford UK; ^3^ Department of Space Science Southwest Research Institute Boulder CO USA; ^4^ Center for Meteorite Studies Arizona State University Tempe AZ USA; ^5^ Smithsonian National Museum of Natural History Washington D C USA; ^6^ Lunar and Planetary Laboratory University of Arizona Tucson AZ USA

**Keywords:** airless bodies, Bennu, laboratory, spectroscopy, thermal infrared

## Abstract

We describe the capabilities, radiometric stability, and calibration of a custom vacuum environment chamber capable of simulating the near‐surface conditions of airless bodies. Here we demonstrate the collection of spectral measurements of a suite of fine particulate asteroid analogs made using the Planetary Analogue Surface Chamber for Asteroid and Lunar Environments (PASCALE) under conditions like those found on Earth and on airless bodies. The sample suite includes anhydrous and hydrated physical mixtures, and chondritic meteorites (CM, CI, CV, CR, and L5) previously characterized under Earth‐ and asteroid‐like conditions. And for the first time, we measure the terrestrial and extra‐terrestrial mineral end members used in the olivine‐ and phyllosilicate‐dominated physical mixtures under the same conditions as the mixtures and meteorites allowing us better understand how minerals combine spectrally when mixed intimately. Our measurements highlight the sensitivity of thermal infrared emissivity spectra to small amounts of low albedo materials and the composition of the sample materials. As the albedo of the sample decreases, we observe smaller differences between Earth‐ and asteroid‐like spectra, which results from a reduced thermal gradient in the upper hundreds of microns in the sample. These spectral measurements can be compared to thermal infrared emissivity spectra of asteroid (101955) Bennu's surface in regions where similarly fine particulate materials may be observed to infer surface compositions.

## Key Points

- Thermal infrared spectra of fine particulate minerals, physical mixtures of those minerals, and meteorites were measured under simulated Bennu conditions
- Comparisons of mineral, physical mixture, and meteorite spectra highlight the spectral behavior when materials are mixed in increasing complexity
- As albedo decreases the spectral effects due to thermal gradients due to the vacuum environment of airless bodies are reduced

## 1 Introduction

The near‐surface environments of Solar System airless bodies including Mercury, the Moon, asteroids, and Mars' moons are controlled by the lack of an appreciable atmosphere, incident solar irradiation, and the compositional and physical properties of the regolith. Early laboratory studies demonstrated that the near‐surface environment, particle size, and porosity affected thermal infrared (TIR) emissivity spectral measurements of terrestrial minerals and lunar soils (e.g., Henderson & Jakosky, 1994; Henderson et al., 1996; Logan & Hunt, 1970; Logan et al., 1973; Salisbury & Walter, 1989). Recent thermal infrared observations of the Moon by the Diviner Lunar Radiometer Experiment on board NASA's Lunar Reconnaissance Orbiter (e.g., Greenhagen et al., 2010; Paige et al., 2010) have allowed advancements in laboratory studies under simulated lunar conditions by combining them with remote sensing observations of the lunar surface. Particularly, Donaldson Hanna et al. (2017) characterized the spectral changes observed in bulk lunar soils resulting from variations in atmospheric pressure and sample heating and compared laboratory spectra with Diviner observations of the Apollo sampling sites. These spectral measurements demonstrated that thermal gradients similar to those in the lunar regolith can be simulated in environment chambers in the laboratory and highlighted additional complexities that arise from differences in albedo, compositional and physical property (e.g., particle size and porosity).

Experience taken from recent lunar‐like laboratory measurements have been applied to other airless bodies including asteroid (101955) Bennu, the target asteroid for NASA's Origins, Spectral Interpretation, Resource Identification, and Security‐Regolith Explorer (OSIRIS‐REx) mission. Donaldson Hanna et al. (2019) measured a suite of physical mixtures and chondritic meteorites thought to be analogous to fine particle materials on Bennu's surface under simulated asteroid conditions. The resulting spectral measurements showed the effects of albedo, where the samples with the lowest albedos (e.g., carbonaceous chondrite meteorites) showed the smallest changes because the low albedo nature of the samples reduced the thermal gradient in the uppermost portion of the sample. Detailed comparisons between asteroid analogs measured in the lab with remote sensing observations from spacecraft like OSIRIS‐REx will better enable the interpretations of current and future thermal infrared remote sensing observations and will further advance laboratory measurements by better constraining the lab conditions that best simulates an airless body's near‐surface conditions. Current and future spacecraft instruments include the Thermal Emission Spectrometer on OSIRIS‐REx mission (OTES) and the Mercury Radiometer and Thermal Infrared Spectrometer (MERTIS) on board ESA's BepiColombo.

Here we describe in detail a new vacuum environment chamber (Planetary Analogue Surface Chamber for Asteroid and Lunar Environments [PASCALE]) capable of simulating the near‐surface environment of Solar System planetary bodies, including a discussion on the calibration and radiometric stability of the experimental set‐up. We then describe the measurement of a suite of primitive asteroid analogs (meteorites, physical mixtures, and terrestrial and extraterrestrial mineral end members) under Earth‐ and Bennu‐like near‐surface conditions and discuss the resulting spectral measurements. The spectral measurements of the fine particulate pure mineral end members used in the physical mixtures are a first of their kind and provide insight into the behaviors of these materials when they are mixed. Insights into the spectral mixing behavior of fine particulates will aid in the interpretation of current and future thermal infrared observations of primitive bodies with fine particulate regolith layers.

## 2 Detailed Description of Experimental Set‐Up

### 2.1 Planetary Analogue Surface Chamber for Asteroid and Lunar Environments

The PASCALE is a vacuum environment chamber capable of simulating the near‐surface conditions of Solar System airless bodies and is located in the Planetary Spectroscopy Facility (PSF) at the University of Oxford. PASCALE was developed and built as an upgrade to Oxford's Simulated Lunar Environment Chamber (SLEC), a vacuum chamber capable of simulating the near surface environments of the Moon and asteroids (Thomas et al., 2012, 2014). While the SLEC experimental set‐up is well characterized and has been used to measure a range of lunar and asteroid analogs, its capabilities are limited (Arnold et al., 2016; Donaldson Hanna et al., 2019; Donaldson Hanna, Thomas, et al., 2012; Donaldson Hanna, Wyatt, et al., 2012; Thomas et al., 2012, 2014). The small size of SLEC only allows for a single sample cup and a calibration target in the chamber at one time, and the inefficient design of its liquid nitrogen (LN_2_) dewar leads to a cooling time of several hours meaning only a single sample and the calibration target can be measured in one day. Thus, the PASCALE experimental set‐up was optimized to reduce the cool down time and increase the number of samples that could be measured under the same environmental conditions.

**Figure 1:**
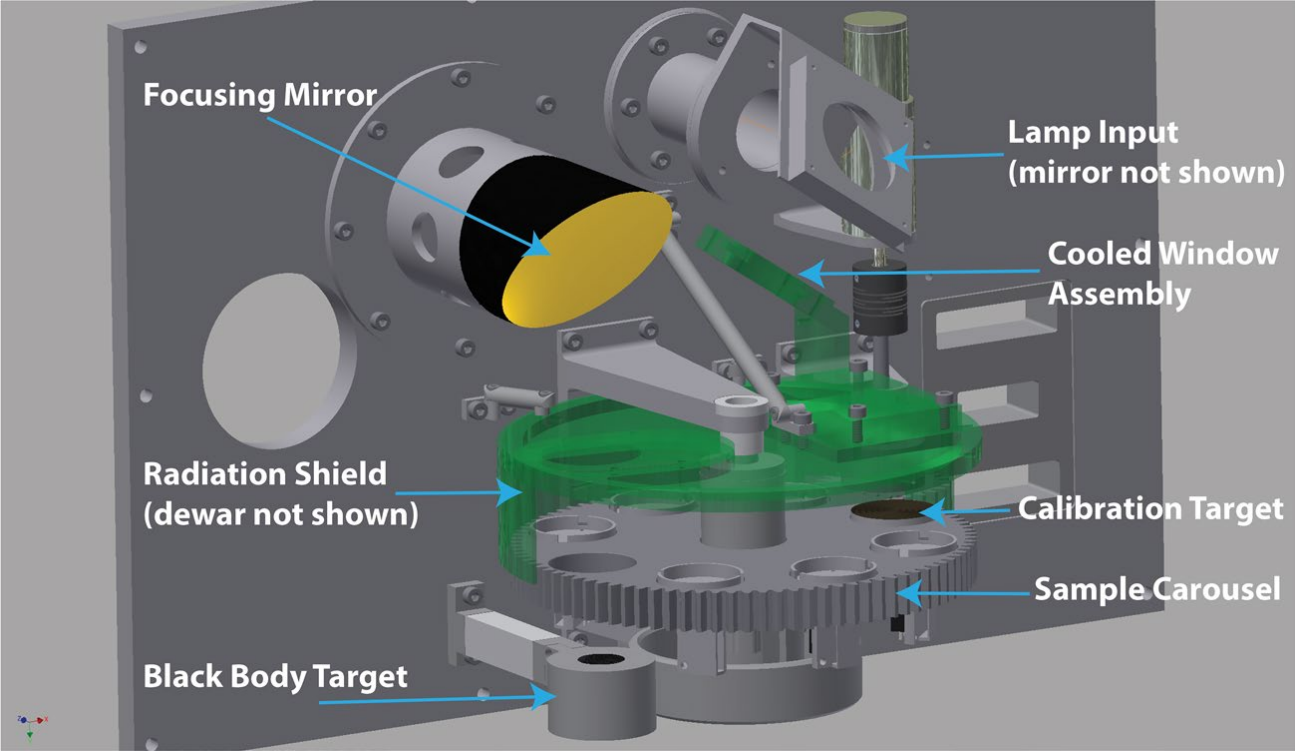
CAD diagram of the interior of PASCALE's chamber with all of the major components identified. PASCALE, Planetary Analogue Surface Chamber for Asteroid and Lunar Environments.

**Figure 2:**
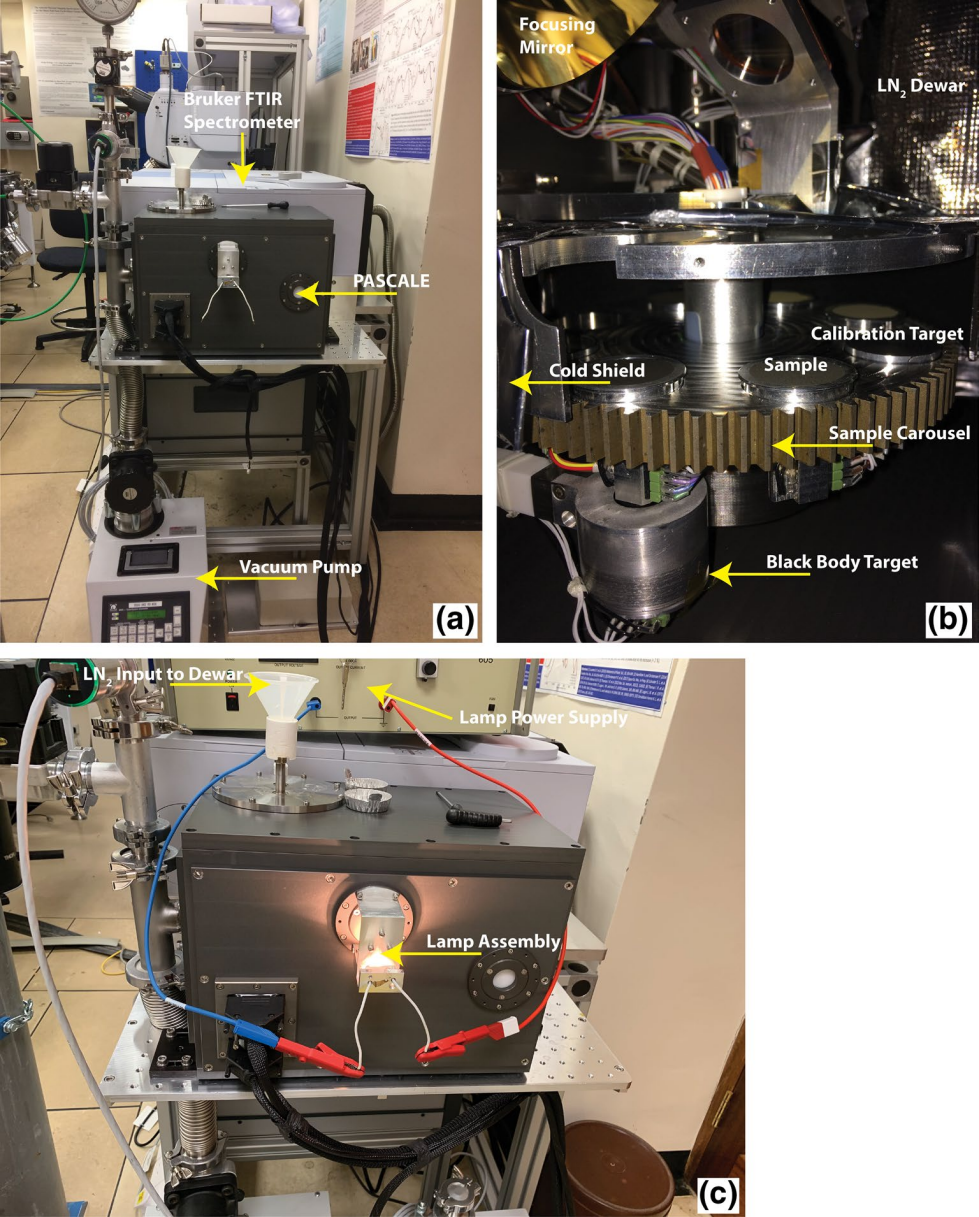
(a) An image of PASCALE attached to the Bruker VERTEX 70V FTIR spectrometer and a turbo vacuum pump. (b) An image of the interior of PASCALE's chamber with all the major components identified. (c) An image of PASCALE with the lamp assembly and dewar input identified. PASCALE, Planetary Analogue Surface Chamber for Asteroid and Lunar Environments.

The PASCALE vacuum chamber (typical operating vacuum of <10^−4^ mbar) consists of a carousel stage enclosed in a cryogen radiation shield, a black body target that sits below the sample carousel, a solar‐like halogen lamp, a dewar, and an off‐axis parabolic mirror (Figures 1 and 2). The sample carousel includes six heated sample cups, a calibration target, and an open slot. The sample carousel can be rotated using a stepper motor controlled using a custom electronics unit to the open slot so that the black body target can be observed. The sample carousel is surrounded by a copper radiation shield that encloses the sample cups and calibration target except for an entrance aperture above the sample cup or calibration target. The radiation shield is coupled to an internally fitted dewar of liquid nitrogen for cooling, coated in Nextel black paint, and surrounded by a blanket of multilayer insulation to ensure it remains isothermal under vacuum. The radiometric calibration of the system is carried out using an internal black body target that is permanently mounted beneath the sample carousel. The black body target is a cylinder ∼20 mm deep with a ridged, interior bottom and is coated in high emissivity Nextel black paint. The black body's circular ridges are 1.5 mm in height and with a spacing of 3.5 mm. The emissivity of the black body is expected to be >0.99 resulting from the black paint coating and multiple blackbody interactions (>1.5 on average) due to the geometry of the ridged target. The calibration target that sits on the sample carousel is similarly ridged and painted with Nextel black paint but lacks the depth of the black body target. As such, measurements of the black body target are used to calibrate the measurements of samples while measurements of the calibration target are used to (1) monitor for contamination of the interior of the chamber and (2) monitor the overall calibration stability of the system by comparing against measurements of the black body. Samples are heated from below using a sample cup heater and/or above using a quartz‐halogen lamp attached to the outside of the chamber and whose beam passes through two 3.0 mm thick, uncoated fused silica windows before hitting a mirror that directs it onto the sample. Because the experiment is designed to test the effects of short wavelength, visible radiation heating, the lamp beam is also passed through a second fused silica window inside the chamber that is heat sunk to the liquid nitrogen dewar, which further reduces the longer wavelength thermal radiation from the lamp. The power of the lamp is controlled with a constant current Bentham 605 power supply, which provides stable and reliable long‐term performance. The temperatures of the system components including the radiation shield, dewar, mirror, sample cups, calibration, and black body targets are measured using embedded Platinum Resistance Thermometer (PRT) 100 temperature sensors. Temperatures are controlled to ± 0.3 K from the set point temperature and logged using a Eurotherm Mini‐8 temperature controller and data logger.

The entire experiment is controlled using a combination of custom control software written in MATLAB and the OPUS Bruker spectrometer software. All measurements are written to a time‐stamped log, including the automated commands saved in native OPUS format, XML self‐describing data files that include all of the set‐up parameters for the spectrometer used to acquire measurements, and simple comma‐separated values (CSV) data point files. The XML files are used by the calibration and analysis software and are time tagged to access the temperature log files at the time of measurement.

Emitted radiation from the sample, black body, or calibration target is collected using an off‐axis parabolic mirror that is heat sunk to the main chamber wall and passed to a Bruker Vertex 70V vacuum Fourier Transform Infrared (FTIR) spectrometer through a cesium iodide (CsI) window. During all measurements the FTIR spectrometer is kept under vacuum pressures (∼2 mbar) and a broadband deuterated triglycine sulfate (DTGS) detector with a response from ∼6,000–50 cm^−1^ and a wide band beam splitter (T240‐T/3) are used for all measurements. We collected 250 interferograms or scans for each sample, black body, and calibration target with a scanner velocity (e.g., rate at which the moving mirror is oscillating) of 1.6 kHz and at a spectral resolution of 4 cm^−1^. The number of interferograms and scanning velocity were chosen to produce spectral measurements with sufficient signal‐to‐noise ratio (SNR) to be able to detect and separate 2% features from the noise, which meets the OSIRIS‐REx mission requirements of analyzing spectral features with ≥5% band depth (Christensen et al., 2018; Lauretta et al., 2017). While we have optimized the FTIR settings to acquire spectral measurements with sufficient SNR to detect 2% features across most of the spectral range, the throughput of Bruker's T240‐T/3 wide band beam splitter between 560 and 620 cm^−1^ is nearly 0 and low albedo samples often have much lower signal across that range. To illustrate this low SNR region of our calibrated spectra, we highlight it with a gray box in all of our spectral figures.

### 2.2 Simulating Near‐Surface Environments in PASCALE

Across the thermal infrared portion of the electromagnetic spectrum, the emitted radiation measured by ground‐ and space‐based telescopes, spacecraft, and in the laboratory originates from the upper hundreds of microns of planetary materials. Laboratory studies have shown that thermal gradients caused by the vacuum environment of an airless body affect the measured emission (e.g., Donaldson Hanna et al., 2017, 2019; Donaldson Hanna, Wyatt, et al., 2012; Henderson & Jakosky, 1994; Logan et al., 1973; Thomas et al., 2012). The PASCALE has the capabilities of simulating a range of planetary near‐surface conditions (i.e., thermal gradients) experienced in the regolith of planetary bodies by controlling the chamber's atmospheric pressure and temperature and the heating of the samples. Here we focus on simulating Earth‐ (ambient) and asteroid‐like conditions. We use ambient measurements to characterize the spectral changes observed in measurements made under simulated asteroid environment (SAE) conditions and to compare against emissivity measurements available in common spectral libraries (e.g., Arizona State University; Christensen et al., 2000).

For ambient conditions, we simulate the approximately isothermal near‐surface environment of the Earth's regolith by backfilling PASCALE with dry, spectrally inactive, nitrogen gas (N_2_) to an atmospheric pressure ∼ 1,000 mbar, maintaining an ambient temperature inside the chamber (∼300 K), and heating samples from below to 353 K. For SAE conditions, we simulate the thermal gradients expected in the near‐surface environment of Bennu, the target asteroid of NASA's OSIRIS‐REx mission, by cooling the vacuum (<10^−4^ mbar) sample chamber to < 125 K using LN_2_, and heating the samples to a maximum brightness temperature of ∼350 K. Brightness temperatures of ∼350 K are achieved by heating samples from below to 333 K and from above using the quartz halogen lamp. These SAE conditions were selected using properties of Bennu described in the OSIRIS‐REx mission's design reference asteroid (Hergenrother et al., 2014) and are similar to the SAE conditions used in Donaldson Hanna et al. (2019). We ensure that samples are measured under a similar thermal environment by adjusting the power of the lamp to heat the surface of each sample to a brightness temperature of approximately 350K (± 3K). We assume that the samples are in thermal equilibrium with their surroundings and istothermal when calculating their brightness temperature although we know that is not the case for SAE measurements. However, the calculated brightness temperatures are still valuable to ensure the thermal stability of the samples during measurements.

### 2.3 Calibration of Spectral Measurements

We are using methodologies described by Ruff et al. (1997) and Thomas et al. (2012) to radiometrically calibrate the PASCALE sample measurements. A typical experiment run includes measurements under ambient and SAE conditions and begins with the measurement of the black body and calibration target followed by the measurement of six samples. We collect three spectral measurements of 250 scans each of each sample at a single temperature, and the black body and calibration target at two temperatures, which in total takes approximately 5 days for both ambient and SAE conditions. We measure the black body and calibration target daily to monitor any changes in the radiometric stability of the PASCALE system. We demonstrate below (Section 2.4) that daily measurements are sufficient for calibration and monitoring of radiometric stability. The black body and calibration target are measured at two temperatures that bracket the sample temperature, typically 340 K (∼67 C) and 360 K (∼87 C). We collect three measurements of the black body at each temperature set point to give an indication of overall random uncertainty in the measurements and three measurements of the calibration target as a further check of stability and to monitor any contamination in the chamber (i.e., if a sample were to release volatiles into the chamber). We also measure each sample three times at its temperature set point to provide an estimate of random measurement uncertainty.

We summarize the calibration procedure below for completeness. We first calculate the instrument response function, *F*, using the black body measurements at two different temperatures (340 K and 360 K in most cases). The instrument response function (Equation 1) allows for the conversion between spectral measurement units (volts, V) to calibrated radiance (Ruff et al., 1997):

(1) $$F=\frac{V_{\text{bb}}\left(T_{1}\right)-V_{\text{bb}}\left(T_{2}\right)}{B_{\text{bb}}\left(T_{1}\right)-B_{\text{bb}}\left(T_{2}\right)}$$

where *V*_bb_ is the voltage of the black body as measured by the FTIR spectrometer and *B*_bb_ is the radiance of a perfect blackbody at the set temperature (*T*) calculated from the Planck function. Two important assumptions are made during this calculation. First, we assume our black body is a “true” black body and has an emissivity ∼1.0, which ensures that any radiation from the environment is not included in the signal recorded by the instrument. Second, we assume that the instrument temperature remains stable throughout the measurement, and its radiance can be estimated using one of the black body measurements to determine the offset in the calibration equation.

The signal (*V*) measured at the FTIR detector is given by Equation 2:

(2) $$V_{\text{meas}}\left(\lambda ,T\right)=\left[\varepsilon_{\text{samp}}\left(\lambda \right)B_{\text{samp}}\left(\lambda ,T\right)+R_{\text{samp}}\left(\lambda \right)\varepsilon_{\text{env}}\left(\lambda \right)B_{\text{env}}\left(\lambda ,T\right)-\varepsilon_{\text{inst}}\left(\lambda \right)B_{\text{inst}}\left(\lambda ,T\right)\right]F$$

where *ε* is the emissivity of the sample (samp), environment (env), and instrument (inst), *R*_samp_ is the reflectance of the sample*, F* is the instrument response function defined in Equation 1, and *B* is the radiance calculated from the Planck function for the sample, environment and instrument. We assume the emissivity of the environmental radiation shield is 1.0, which is a good approximation for PASCALE as the cold radiation shield will have a limited contribution to the emitted radiation and whatever signal may be added is blackbody in nature. The contribution to the measured signal due to the spectrometer radiance is estimated using the black body measurements at the higher set point temperature and Equation 3:

(3) $$\varepsilon_{\text{inst}}B_{\text{inst}}=B_{\text{bb}}-\frac{V_{\text{bb}}}{F}$$

Finally, we calculate the emissivity of the measured sample using Equation 4:

(4) $$\varepsilon_{\text{samp}}=\frac{\frac{V_{\text{meas}}}{F}-B_{\text{env}}+B_{\text{inst}}}{B_{\text{samp}}-B_{\text{env}}}$$

A critical step in the Ruff et al. (1997) calibration methodology is the determination of the sample temperature for converting from calibrated radiance to emissivity. To allow *B*_samp_ to be estimated, a point in the spectrum is required where the emissivity can be assumed to be unity (or an independently known emissivity value). The typical approach is to convert the measured sample voltage (*V*_meas_) into radiance and then calculate the equivalent brightness temperature per wavenumber. The wavenumber having the maximum brightness temperature, within a defined spectral range, is then taken to have unit emissivity and the sample temperature can be estimated. For silicate‐dominated samples, the maximum brightness temperature typically coincides with the Christiansen feature (CF), a spectral feature known to be related to mineralogy (Conel, 1969). For samples with more complex compositions like carbonaceous chondrite meteorites, the maximum brightness temperature determination can be complicated by multiple, broad maxima in the brightness temperature spectrum (Salisbury et al., 1991). Positive and negative slopes are the typical artifacts introduced into calibrated emissivity spectra when an incorrect sample temperature is used in the emissivity calculation or when the instrument or environmental temperatures vary during measurement. However, the positions and shapes of spectral features should be unchanged.

We use the Thomas et al. (2012) approach to calculate the uncertainties in our laboratory emissivity spectra using repeat measurements of the samples, black body, and calibration target. The uncertainty in the instrument response function, *F*, is calculated using repeat measurements of the black body and Equation 5.

(5) $$\Delta F\left(\lambda \right)=\frac{1}{B_{\text{bb}}\left(\lambda ,T_{1}\right)-B_{\text{bb}}\left(\lambda ,T_{2}\right)\;}\sqrt{{\left(\Delta V_{\text{bb}}\left(\lambda ,T_{1}\right)\right)}^{2}+{\left(\;\Delta V_{\text{bb}}\left(\lambda ,T_{2}\right)\right)}^{2}}$$

We then calculate the resulting uncertainty in the emissivity measurement of the sample using repeat measurements of the black body sample and Equation 6.

(6) $$\Delta \varepsilon_{\text{samp}}\left(\lambda \right)=\sqrt{{\left(\left(\frac{V_{\text{bb}}\left(\lambda ,T_{2}\right)}{F}\right)\sqrt{{\left(\frac{\Delta V_{\text{bb}}\left(\lambda ,T_{2}\right)}{V_{\text{bb}}\left(\lambda ,T_{2}\right)}\right)}^{2}+\;{\left(\frac{\Delta F}{F}\right)}^{2}}\right)}^{2}+{\left(\;\left(\frac{V_{\text{meas}}\left(\lambda ,T\right)}{F}\right)\sqrt{{\left(\frac{\Delta V_{\text{meas}}\left(\lambda ,T\right)}{V_{\text{meas}}\left(\lambda ,T\right)}\right)}^{2}+{\left(\frac{\Delta F}{F}\right)}^{2}}\right)}^{2}}$$

**Figure 3:**
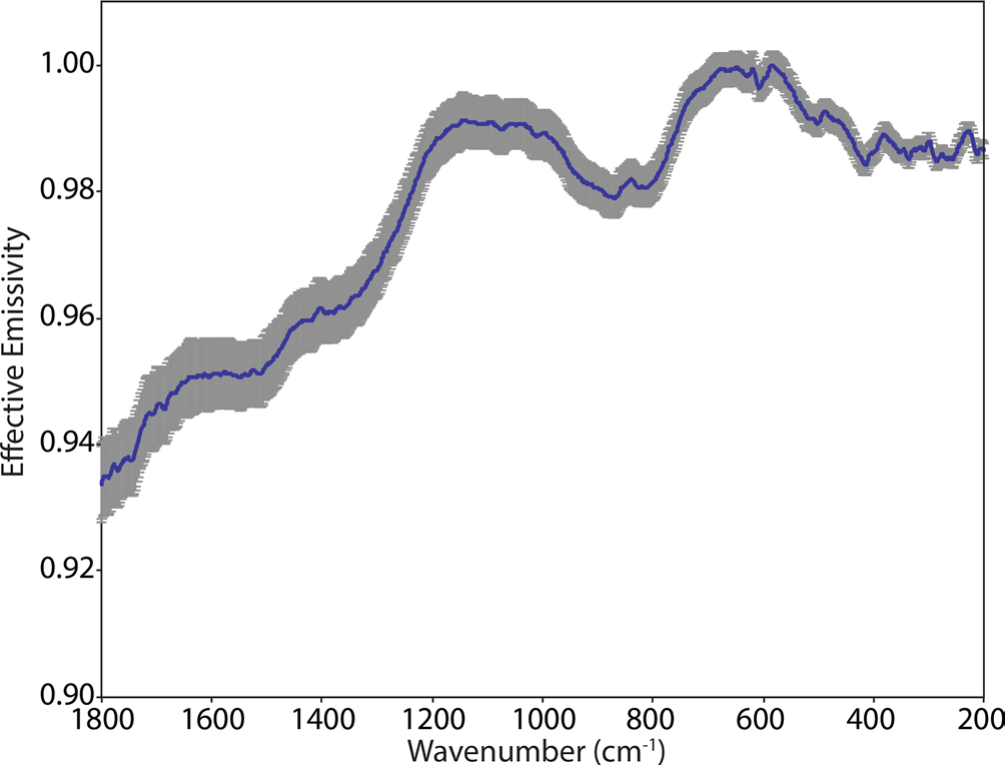
Average ambient emissivity spectrum of Murchison with the uncertainty in the measurements expressed as error bars on the average spectrum.

Figure 3 shows an ambient emissivity measurement of the Murchison meteorite (Donaldson Hanna et al., 2019) with the uncertainties in emissivity included as error bars. We chose to show the uncertainties for Murchison as it is a low albedo material that shows only small deviations from unit emissivity and has a low signal‐to‐noise ratio across the thermal infrared spectral range (e.g., average emissivity of 0.98 across the 1,200–200 cm^−1^ spectral range). Thus, we are showing a case with the highest uncertainties in the emissivity measurements.

### 2.4 Simulating Near‐Surface Environments in PASCALE

We use calibration target measurements collected from March 2019 through May 2019 (*N* = 54) to demonstrate the radiometric stability and overall performance of the PASCALE system (here we mean the environment chamber plus the FTIR spectrometer). The most sensitive measure of the system's radiometric stability and temperature reproducibility is to compare each raw detector measurement of the calibration target to the long‐term mean of all the measurements to date. Comparisons are calculated using Equation 7:

(7) $$\%\text{diff}=\frac{V_{\text{cal}}-V_{\text{cal\_avg}}}{V_{\text{cal\_avg}}}\times 100$$

**Figure 4:**
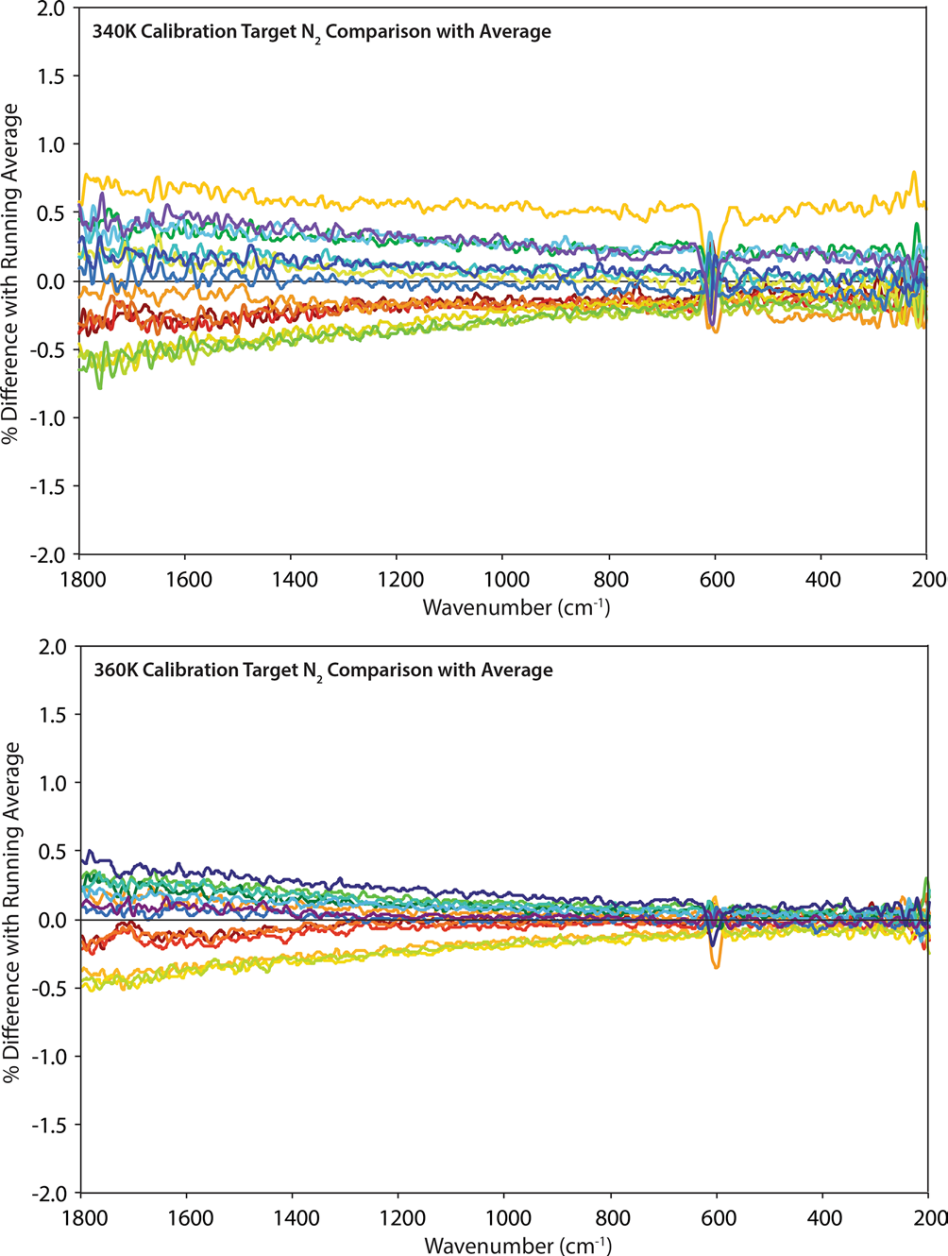
Comparison between individual measurements of the calibration target at 340 K (top) and 360 K (bottom) and the long‐term average to demonstrate the spread in measurements under ambient conditions. Measurements are plotted in order of time starting with red and ending with purple.

**Figure 5:**
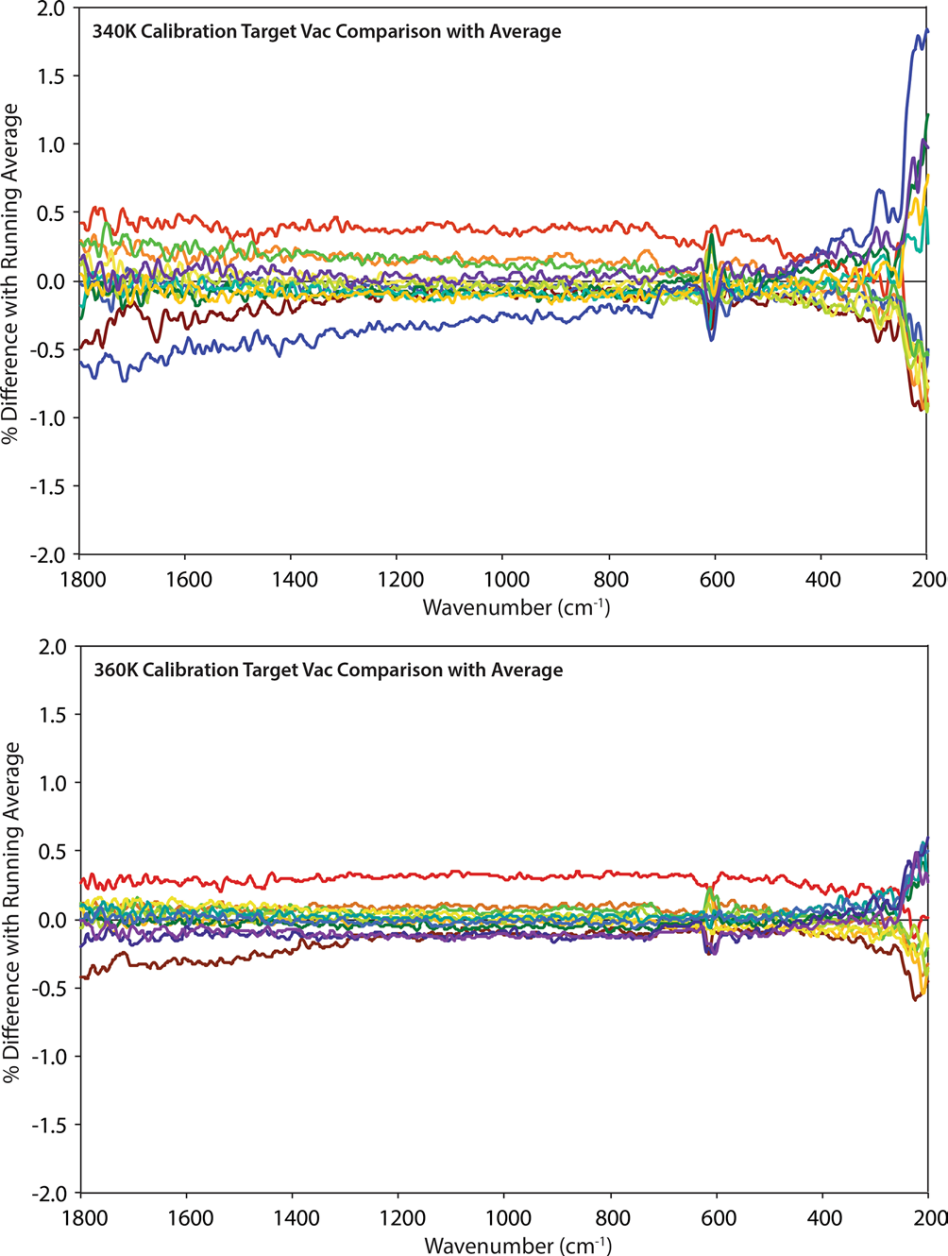
Comparison between individual measurements of the calibration target at 340 K (top) and 360 K (bottom) and the long‐term average to demonstrate the spread in measurements under SAE conditions. Measurements are plotted in order of time starting with red and ending with purple. SAE, simulated asteroid environment.

The raw measurements are grouped by calibration target set point temperature (e.g., 340 or 360 K) and environmental conditions (e.g., ambient or SAE conditions). Ambient measurements at 340 K and 360 K show spreads on average of ± 0.75% and ± 0.5%, respectively, across the 1,800–200 cm^−1^ spectral range with the largest deviations at higher frequencies where the flux is at the lowest (see Figure 4). SAE measurements at 340 K and 360 K show similar spreads of ± 0.5% across the 1,800–200 cm^−1^ spectral range with the largest deviations at lower frequencies where the emissivity of the calibration target drops off (see Figure 5). Thus, across a 3‐month period the combined environment chamber and spectrometer demonstrate a radiometric stability of ≤ 0.75% under ambient and SAE conditions.

**Figure 6:**
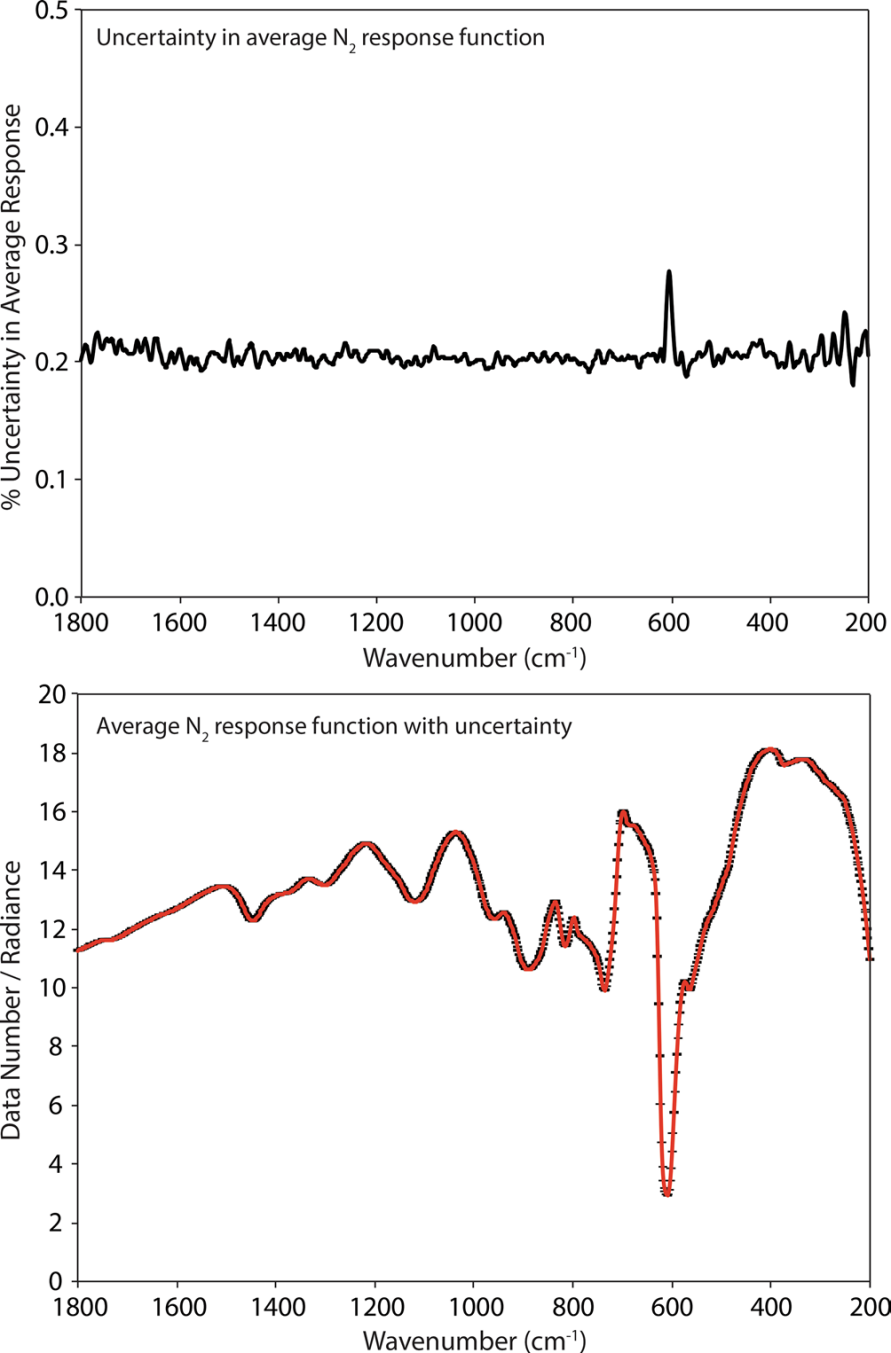
The uncertainty in the mean ambient instrument response function (top) and the average ambient instrument response function with the uncertainty envelope (bottom). The significant drop in radiance near 600 cm^−1^ is due to the beamsplitter's low throughput in this spectral range.

**Figure 7:**
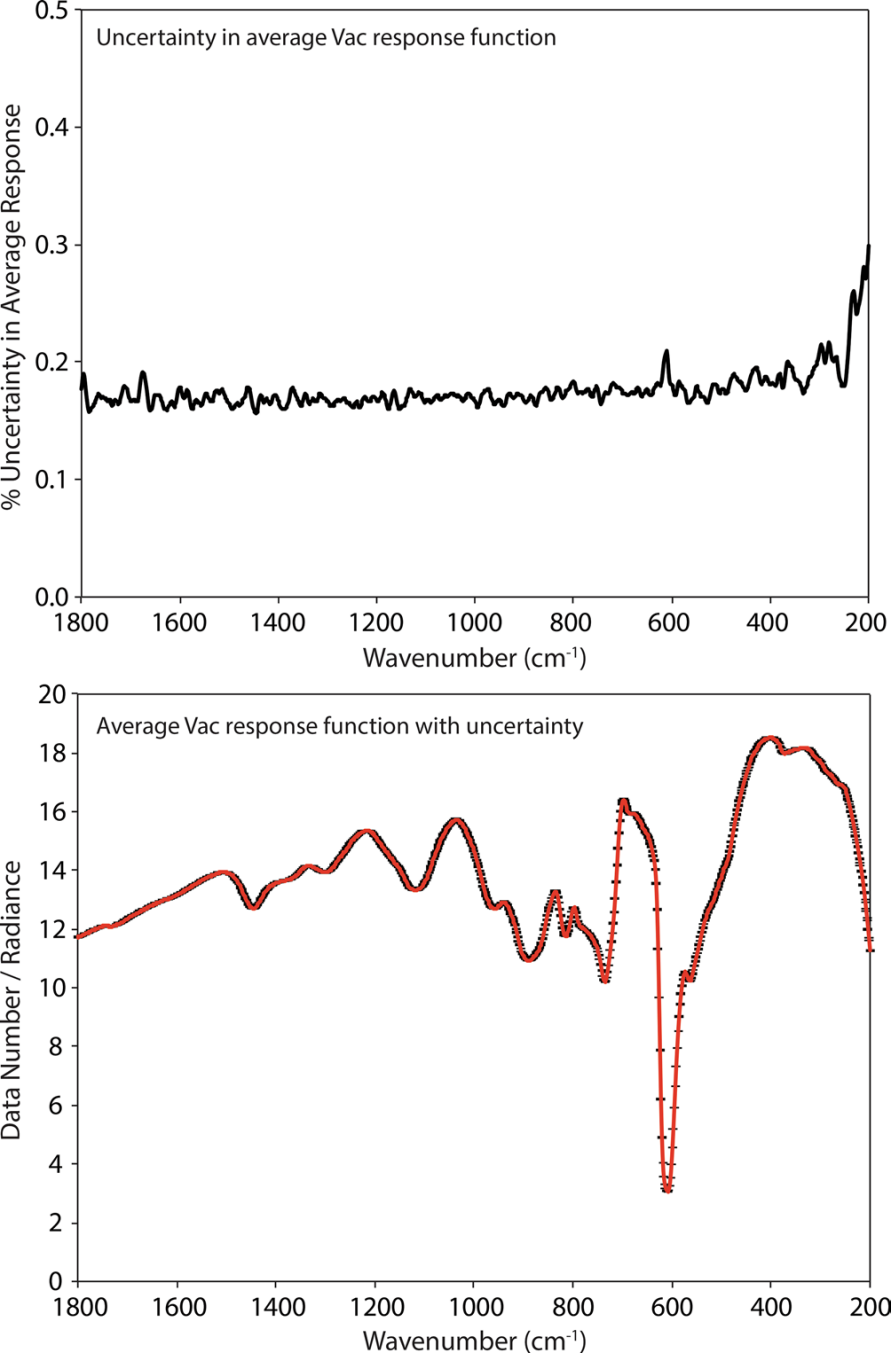
The uncertainty in the mean SAE instrument response function (top) and the average SAE instrument response function with the uncertainty envelope (bottom). SAE, simulated asteroid environment.

We can also use the time series of raw calibration target measurements to estimate the uncertainty in the instrument response function, *F* (Equation 1), for both ambient and SAE measurements. Note that due to the emissivity of the calibration target being only ∼0.99 this estimation only tracks the variation in the system response. The uncertainty in the average ambient instrument response function is ∼0.25% across the 1,800–200 cm^−1^ spectral range (see Figure 6). The increase in uncertainty near 600 cm^−1^ is due to the low throughput of the wide band beam splitter as can be seen in the average instrument response function in Figure 6. The uncertainty in the average SAE instrument response function is ∼0.18% across the 1,800–200 cm^−1^ spectral range (see Figure 7). Again, the increases in uncertainty near 600 cm^−1^ and at lower frequencies (<300 cm^−1^) are due to the low throughput of the beam splitter and the roll‐off in the emissivity of Nextel paint, respectively. Thus, across a 3‐month period the instrument response function was stable to ≤0.25% under ambient and SAE conditions.

## 3 Suite of Analog Samples

We measured a suite of physical mixtures consisting of terrestrial and extra‐terrestrial minerals as well as chondritic meteorites that were previously characterized as part of the OSIRIS‐REx blind test (Donaldson Hanna et al., 2019). Five of the physical mixtures were made with minerals and their abundances consistent with those found in type 3 chondrites (i.e., olivine, pyroxene, plagioclase feldspar, Fe‐metal, and troilite), and with minor amounts (<5%) of phyllosilicates, carbonates, and synthetic insoluble organic matter (IOM). The other five physical mixtures were made with minerals and their abundances consistent with type 1 and 2 aqueously altered carbonaceous chondrites (i.e., phyllosilicates, troilite, and magnetite), and with minor amounts of olivine, carbonate, and IOM. The chondritic meteorites include Allende (CV3_OxA_; USNM 3529‐21), Farmington (L5 shock blackened; USNM 5856), Murchison (CM2; USNM 5453), MIL 090001 (CR2; split 45), ALH 83100 (CM1/2; split 282), Orgueil (CI; USNM 6476), and Vigarano (CV3_red_; USNM 3137). In addition, we measured most of the terrestrial and extra‐terrestrial minerals used in the physical mixtures and terrestrial minerals commonly identified in carbonaceous chondrite meteorites. These minerals include San Carlos olivine (NMNH 170992), Mono Lake saponite (NMNH 154236), Minas Gerais magnetite (NMNH 114111), New Mexico calcite (NMNH 143299‐18), Johnstown orthopyroxene (USNM 6633 and USNM 7418), Kakanui augite (NMNH 122142), Mundrabilla troilite (USNM 914), spinel (WAR‐0687), Santa Eulalia pyrrhotite (VEH‐1), and Harz Mountains cronstedtite (CC‐1).

The physical mixtures and chondritic meteorites were crushed, sieved, and mixed to have particle size distributions representative of asteroid regolith formed through impact gardening and similar to the fine particulate nature of the Itokawa samples (Cintala & Hörz, 2008; Donaldson Hanna et al., 2019; Flynn et al., 2009; Nakamura et al., 2011). The terrestrial and extra‐terrestrial mineral end members were crushed and sieved using an agate mortar and pestle to particle size fractions of <90 μm. The one exception is the spinel sample, which was obtained already prepared for a separate study at <45 μm. We include the terrestrial and extra‐terrestrial mineral samples, their origin, and their respective particle size fractions in Table 1.

**Table 1:** Terrestrial and Extra‐Terrestrial Minerals Spectrally Characterized

Mineral	Mineral type	Source	Particle size fraction (mm)	Physical mixture end member
Augite	Silicate	Kakanui, New Zealand	< 90	Yes
Olivine	Silicate	San Carlos, Arizona, USA	< 90	Yes
Orthopyroxene	Silicate	Johnstown Meteorite	< 90	Yes
Cronstedtite	Phyllosilicate	Genrode, Harz Mountains, Saxony, Germany	< 90	–
Saponite	Phyllosilicate	Mono Lake, California, USA	< 90	Yes
Calcite	Carbonate	New Mexico, USA	< 90	Yes
Magnetite	Oxide	Minas Gerais, Brazil, South America	< 90	Yes
Spinel	Oxide	Ward's Minerals	< 45	–
Troilite	Sulfide	Mundrabilla Meteorite	< 90	Yes
Pyrrhotite	Sulfide	Santa Eulalia, Chihuahua, Mexico	< 90	–

### 3.1 Sample Preparation for Measurements

**Figure 8:**
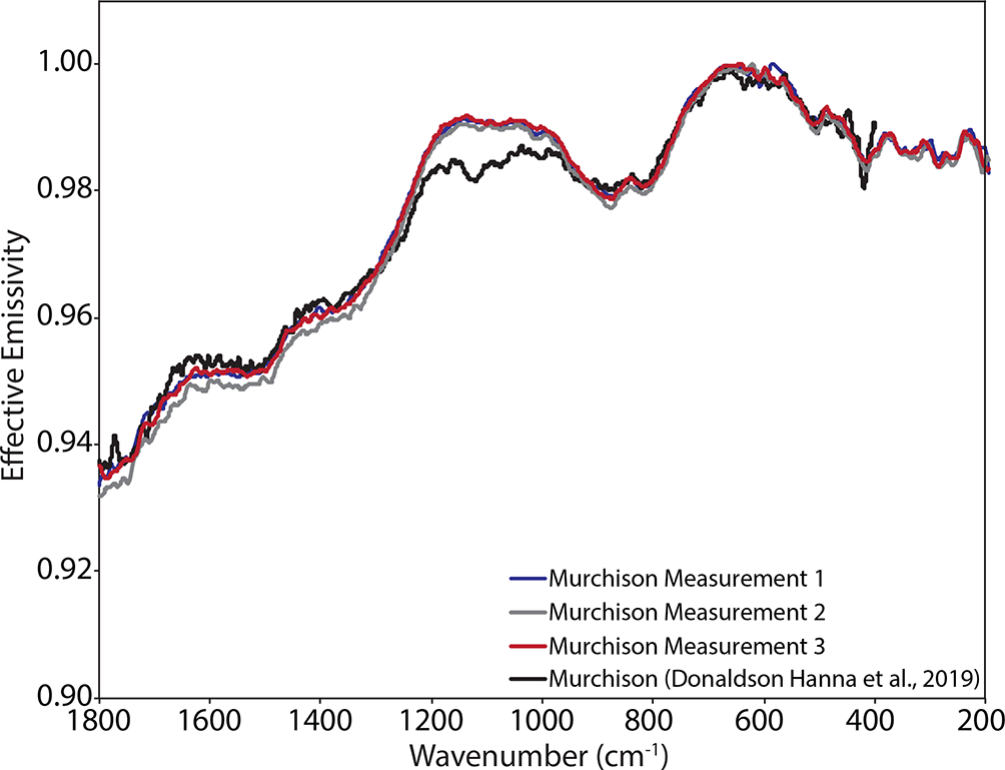
Ambient spectral measurements of Murchison made in PASCALE on separate days and in the Simulated Lunar Environment Chamber (SLEC; Donaldson Hanna et al., 2019). For each measurement, the Murchison sample was prepared in the sample cup in the same way. PASCALE spectra are red, green and blue and the SLEC spectrum is in black. PASCALE, Planetary Analogue Surface Chamber for Asteroid and Lunar Environments.

For the spectral measurements, we prepared each of the samples in a repeatable manner to minimize variation in the physical condition of the sample. We spooned sample material into each sample cup and then drew the flat end of a stainless steel spatula across the surface of the sample. This technique creates a nearly flat surface without compressing the upper most layer, which can alter the spectral contrast of features across the thermal infrared (e.g., Salisbury & Wald, 1992). This is the same sample preparation technique used by Donaldson Hanna et al. (2019) and by other spectroscopy laboratories (Greenhagen et al., 2020; Maturilli et al., 2006, 2008), thus can be easily compared to other thermal infrared spectral measurements. To demonstrate the repeatability of the sample preparation technique, we measured the Murchison sample on three separate occasions in PASCALE. We can then compare the resulting spectra to one another as well as with the spectral measurements of the same Murchison sample measured in the Simulated Lunar Environment Chamber (SLEC; Donaldson Hanna et al., 2019). As seen in Figures 3 and 8, the observed differences between the repeat ambient measurements of Murchison are smaller than the uncertainties in the ambient emissivity measurement. Comparing the SLEC ambient spectrum to the PASCALE ambient spectra, all the main spectral features are identified at the same frequencies and the spectral contrast of most spectral features is similar. However, we do observe subtle differences (<1%) in the slope of the emissivity peak between ∼1,200 and 1,000 cm^−1^. These subtle differences could result from differences in the sample preparation and/or the calibration between the two environment chambers.

### 3.2 Analysis of Spectral Measurements

We describe the thermal infrared spectral measurements by identifying diagnostic features and quantifying the observed changes between measurements made under ambient and SAE conditions. The diagnostic thermal infrared spectral features we identify are the CF, the fundamental vibrational bands or reststrahlen bands (RB), and the transparency feature (TF). These spectral features have been shown to be indicative of bulk composition of rocks and meteorites and the mineralogy for silicate minerals (e.g., Conel, 1969; Cooper et al., 2002; Hamilton, 2000; Salisbury et al., 1991; Salisbury & Walter, 1989). The position (e.g., frequency) of each spectral feature is identified by finding the maximum (e.g., CF) or minimum (e.g., RB and TF) in a smoothed emissivity spectrum. Full resolution laboratory spectra are smoothed with a boxcar average of sufficient width (typically 9 wavenumber bins) to reduce noise in spectra without changing the position of diagnostic features. Previous studies have shown that similar techniques can identify the position of spectral features with an accuracy of ± 3 cm^−1^ (e.g., Donaldson Hanna, Thomas, et al., 2012; Thomas et al., 2012). We also quantify the spectral contrast (e.g., difference in emissivity) between the CF and the first RB to highlight changes due to environmental conditions, such as thermal gradients (e.g., Donaldson Hanna et al., 2017; Donaldson Hanna, Thomas, et al., 2012; Donaldson Hanna, Wyatt, et al., 2012; Henderson & Jakosky, 1994; Henderson et al., 1996; Logan & Hunt, 1970; Logan et al., 1973; Salisbury & Walter, 1989; Shirley & Glotch, 2019).

## 4 Results

**Figure 9:**
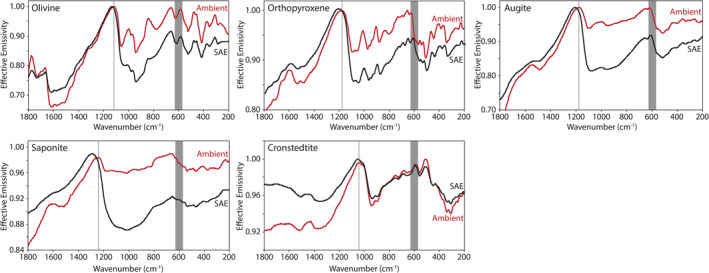
Ambient and SAE anhydrous and hydrated silicate spectra. The gray vertical lines highlight the Christiansen feature (CF) position identified in the ambient spectra. The gray shaded regions highlight the low signal‐to‐noise ratio (SNR) regions in the spectra due to the FTIR’s widerange beam splitter. FTIR, Fourier Transform Infrared; SAE, simulated asteroid environment.

**Figure 10:**
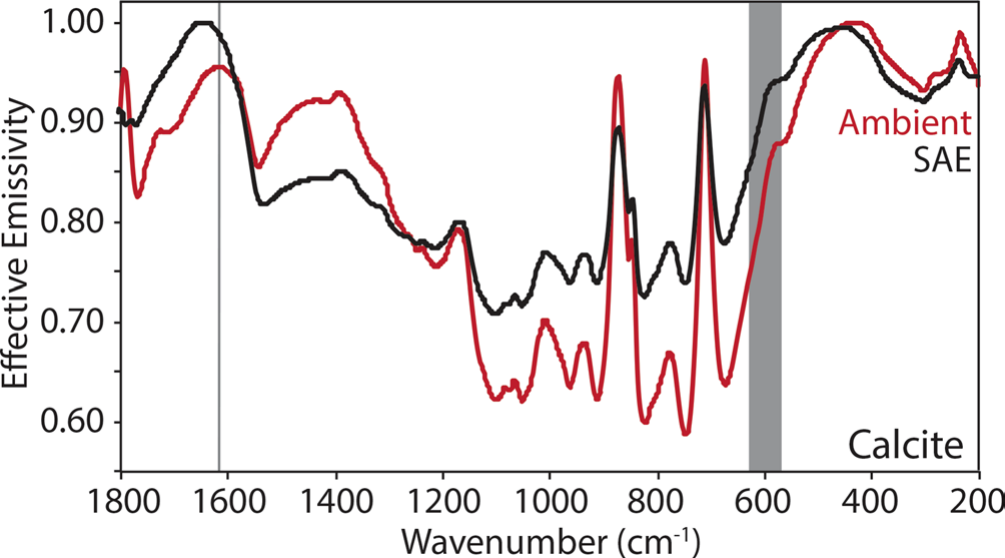
Ambient and SAE carbonate spectra. The gray vertical line highlights the CF position identified in the ambient spectrum. The gray shading highlights the low SNR region in the spectrum due to the FTIR’s widerange beam splitter. CF, Christiansen feature; FTIR, Fourier Transform Infrared; SAE, simulated asteroid environment; SNR, signal‐to‐noise ratio.

**Figure 11:**
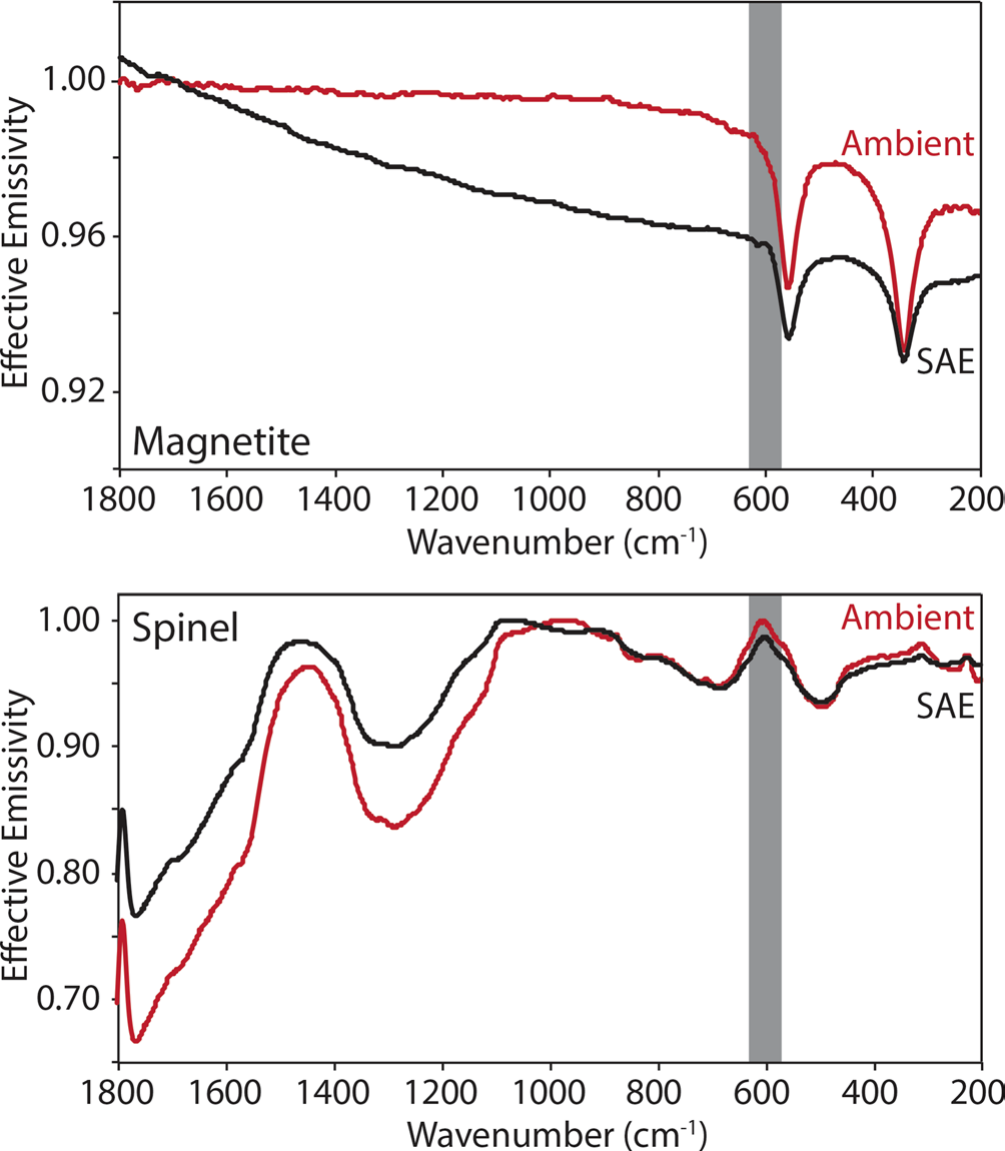
Ambient and SAE oxides spectra. The gray vertical lines highlight the CF position identified in the ambient spectra. The gray shaded regions highlight the low SNR regions in the spectra due to the FTIR’s widerange beam splitter. CF, Christiansen feature; FTIR, Fourier Transform Infrared; SAE, simulated asteroid environment; SNR, signal‐to‐noise ratio.

**Figure 12:**
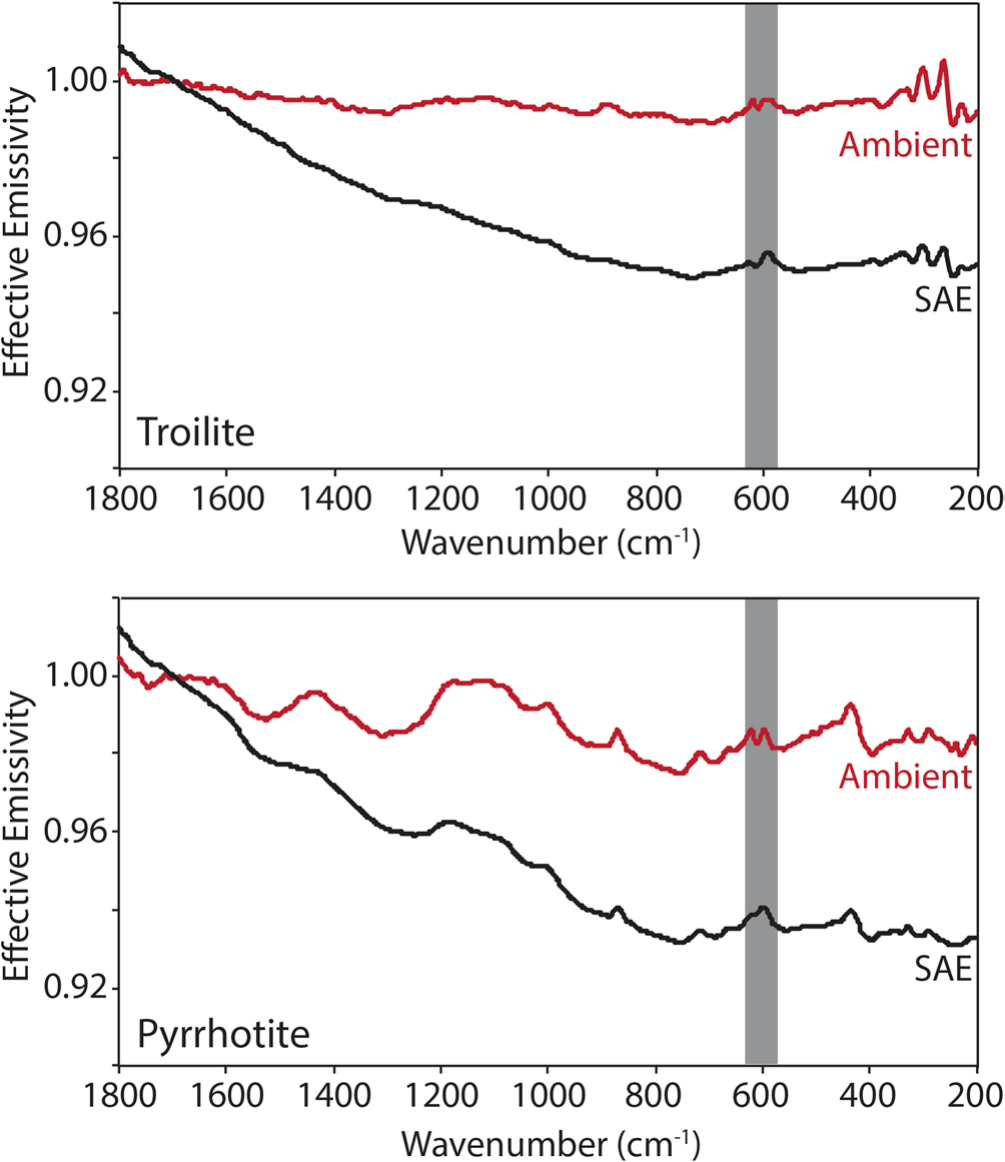
Ambient and SAE sulfides spectra. The gray vertical lines highlight the CF position identified in the ambient spectra. The gray shaded regions highlight the low SNR regions in the spectra due to the FTIR’s widerange beam splitter. CF, Christiansen feature; FTIR, Fourier Transform Infrared; SAE, simulated asteroid environment; SNR, signal‐to‐noise ratio.

**Figure 13:**
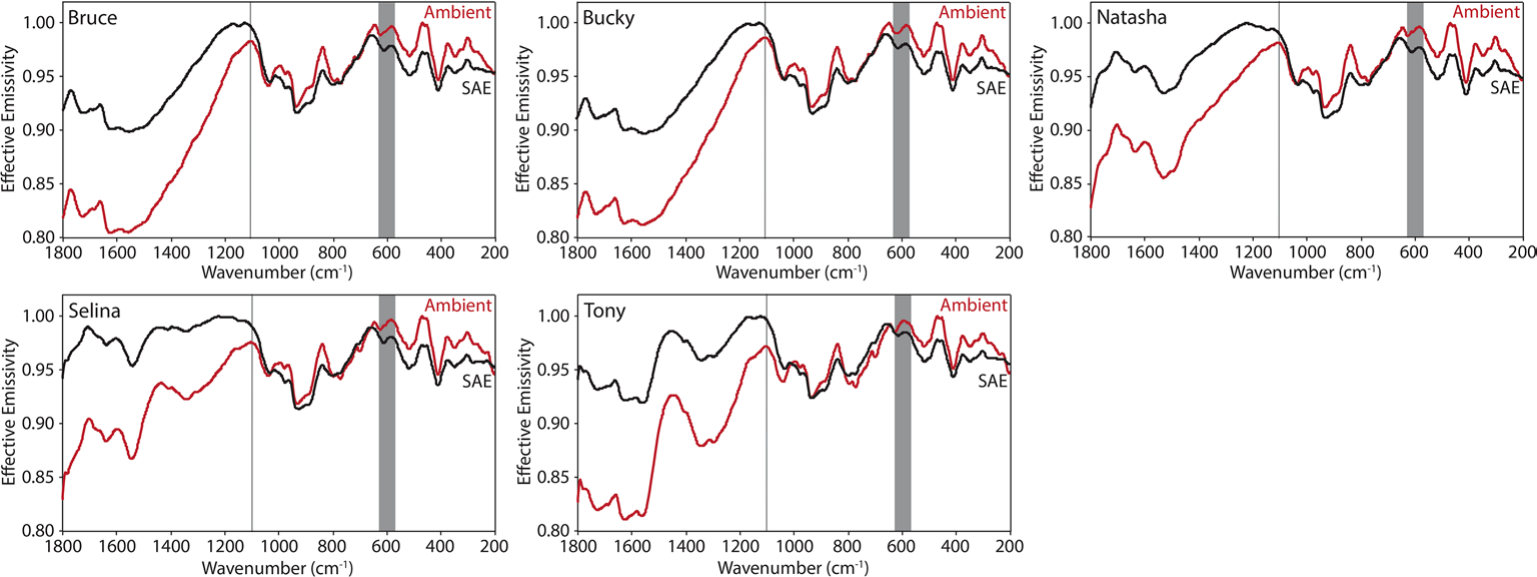
Ambient and SAE spectra of the olivine‐rich physical mixtures. The gray vertical lines highlight the CF position identified in the ambient spectra. The gray shaded regions highlight the low SNR regions in the spectra due to the FTIR’s widerange beam splitter. CF, Christiansen feature; FTIR, Fourier Transform Infrared; SAE, simulated asteroid environment; SNR, signal‐to‐noise ratio.

**Figure 14:**
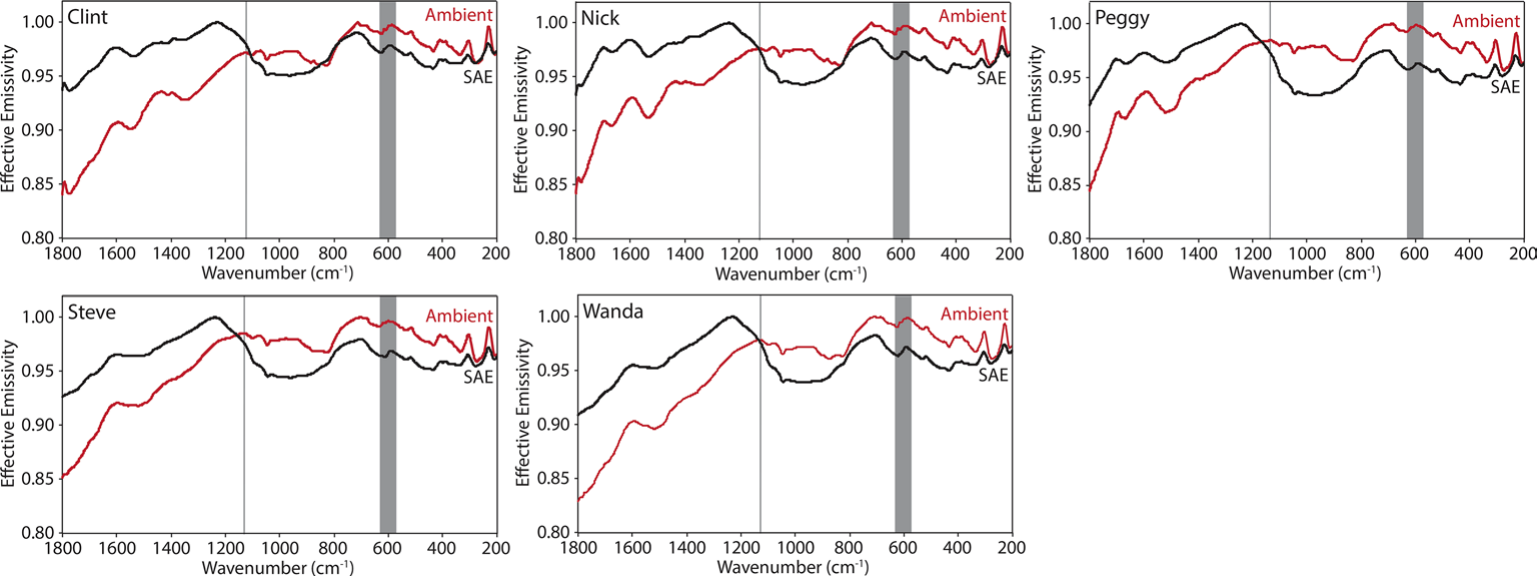
Ambient and SAE spectra of the phyllosilicate‐rich physical mixtures. The gray vertical lines highlight the CF position identified in the ambient spectra. The gray shaded regions highlight the low SNR regions in the spectra due to the FTIR’s widerange beam splitter. CF, Christiansen feature; FTIR, Fourier Transform Infrared; SAE, simulated asteroid environment; SNR, signal‐to‐noise ratio.

**Figure 15:**
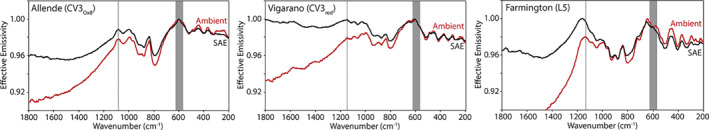
Ambient and SAE spectra of the anhydrous meteorites. The gray vertical lines highlight the CF position identified in the ambient spectra. The gray shaded regions highlight the low SNR regions in the spectra due to the FTIR’s widerange beam splitter. CF, Christiansen feature; FTIR, Fourier Transform Infrared; SAE, simulated asteroid environment; SNR, signal‐to‐noise ratio.

**Figure 16:**
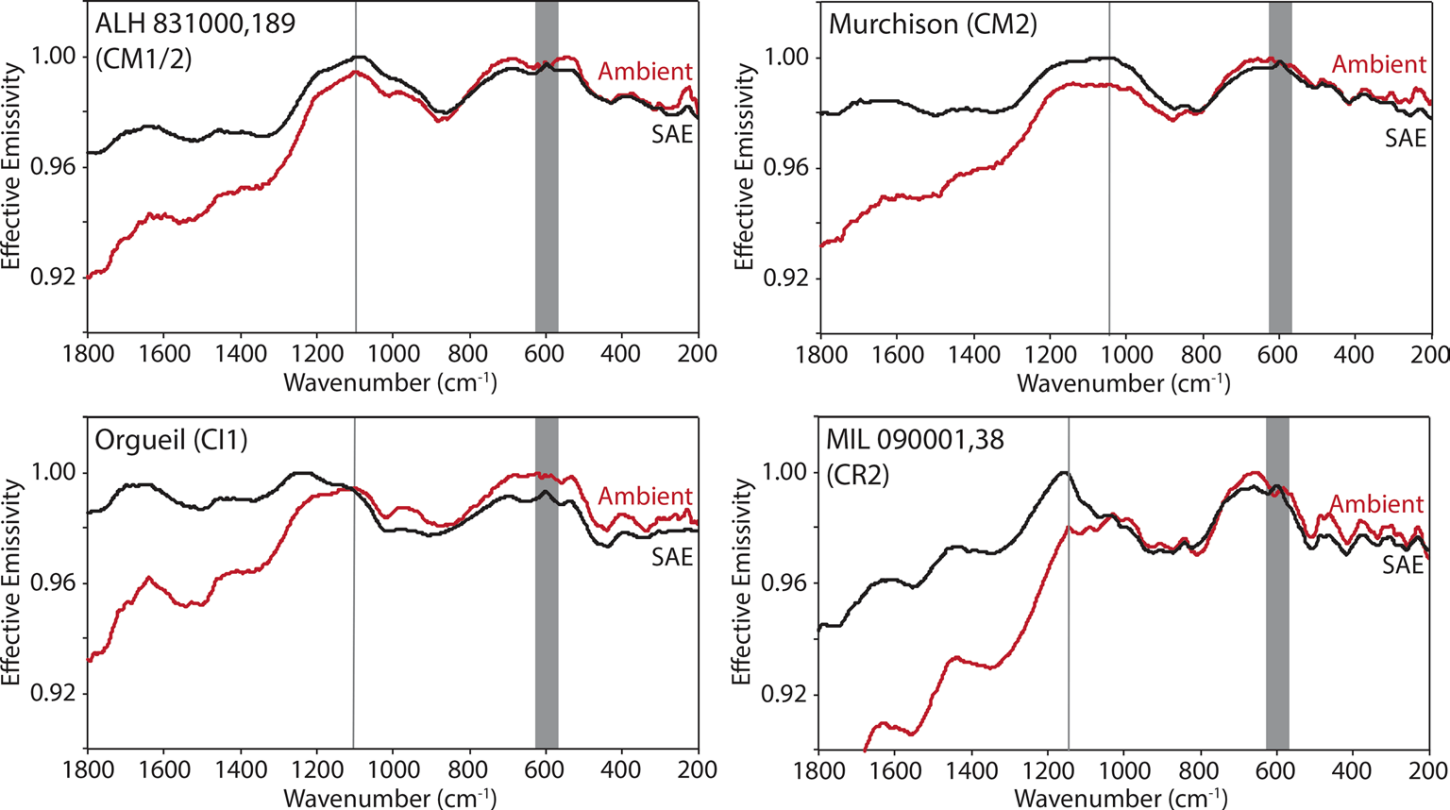
Ambient and SAE spectra of the hydrated meteorites. The gray vertical lines highlight the CF position identified in the ambient spectra. The gray shaded regions highlight the low SNR regions in the spectra due to the FTIR’s widerange beam splitter. CF, Christiansen feature; FTIR, Fourier Transform Infrared; SAE, simulated asteroid environment; SNR, signal‐to‐noise ratio.

Here we describe the thermal infrared emissivity measurements made under ambient and simulated asteroid environment conditions of the terrestrial and extra‐terrestrial mineral, the physical mixtures primarily composed of those minerals, and the chondritic meteorites. Ambient and SAE spectra are presented in Figures 9, 10, 11, 12, 13, 14, 15, 16 and the frequencies of spectral features and the spectral contrasts between the CFs and RBs are presented in Tables 2, 3, 4.

**Table 2:** Features (in cm^−1^) Identified in Terrestrial and Extra‐Terrestrial Mineral Spectra

Name	CF1	CF2	RB1	RB2	RB3	RB4	RB5	RB6	RB7	RB8	RB9	RB10	TF 1	CF emiss	RB1 emiss	Contrast
Ambient measurements																
Augite	1176.1	620.9	1099.0	946.7	512.9	403.0	335.5	–	–	–	–	–	–	1.0000	0.9389	6.1
Olivine	1118.3	659.4	1056.6	979.5	940.9	624.7	522.5	466.6	414.6	352.9	283.5	–	777.0	0.9999	0.8655	13.4
Orthopyroxene	1176.1	624.7	1091.3	971.7	954.4	879.2	566.9	541.8	507.1	443.5	385.6	339.4	802.1	0.9997	0.9165	8.3
Cronstedtite	1045.0	509.0	940.9	898.5	726.9	559.2	451.2	339.4	308.5	–	–	–	–	0.9960	0.9487	4.7
Saponite	1245.5	659.4	1176.1	1018.0	536.0	462.8	374.1	–	–	–	–	–	–	0.9940	0.9726	2.1
Calcite	1617.6	–	1544.3	873.4	711.5	–	–	–	–	–	–	–	–	0.9552	0.8553	10.0
Magnetite	–	–	557.2	343.2	–	–	–	–	–	–	–	–	–	–	–	–
Spinel	–	–	1289.9	690.3	497.5	–	–	–	–	–	–	–	–	–	–	–
SAE measurements																
Augite	1207.0	609.3	1075.9	958.2	514.8	412.6	337.4	–	–	–	–	–	–	1.0000	0.8141	18.6
Olivine	1131.8	661.3	1031.5	979.5	940.9	617.0	522.5	466.6	414.6	352.9	285.4	–	777.0	0.9999	0.8005	19.9
Orthopyroxene	1214.7	617.0	1091.3	971.7	954.4	881.1	564.9	541.8	507.1	445.4	385.6	341.3	817.5	1.0000	0.8545	14.6
Cronstedtite	1052.7	511.0	937.0	896.6	728.8	557.2	449.3	337.4	308.5	–	–	–	–	1.0000	0.9566	4.3
Saponite	1289.9	653.6	1087.4	1012.2	534.1	464.7	381.8	–	–	–	–	–	–	1.0000	0.8857	11.4
Calcite	1650.4	–	1534.7	873.4	713.4	–	–	–	–	–	–	–	–	1.0000	0.8175	18.3
Magnetite	–	–	557.2	343.2		–	–	–	–	–	–	–	–	–	–	–
Spinel	–	–	1289.9	684.5	497.5	–	–	–	–	–	–	–	–	–	–	–

Abbreviations: CF, Christiansen feature; RB, reststrahlen bands; SAE, simulated asteroid environment; TF, transparency feature.

**Table 3:** Features (in cm^−1^) Identified in Physical Mixture Spectra

Name	CF1	CF2	RB1	RB2	RB3	RB4	RB5	RB6	RB7	RB8	RB9	TF 1	TF 2	CF emiss	RB1 emiss	Contrast
Ambient measurements																
Olivine‐rich physical mixtures																
Bruce	1104.8	647.8	1039.2	977.5	937.0	624.7	518.7	460.8	412.6	349.0	271.9	796.3	773.2	0.9830	0.9415	4.2
Bucky	1104.8	647.8	1039.2	977.5	933.2	620.9	518.7	460.8	412.6	349.0	273.8	796.3	773.2	0.9866	0.9478	3.9
Natasha	1108.6	647.8	1039.2	977.5	935.1	626.6	518.7	460.8	412.6	349.0	277.7	794.4	773.2	0.9818	0.9432	3.9
Selina	1102.8	645.9	1039.2	979.5	931.3	626.6	518.7	460.8	410.7	350.9	275.7	796.3	773.2	0.9760	0.9447	3.1
Tony	1104.8	645.9	1041.1	979.5	937.0	626.6	518.7	460.8	410.7	349.0	275.7	796.3	773.2	0.9726	0.9395	3.3
Phyllosilicate‐rich physical mixtures																
Clint	1122.1	713.4	1102.8	1046.9	885.0	859.9	624.7	534.1	433.8	337.4	273.8	827.1	–	0.9719	0.9698	0.2
Nick	1126.0	711.5	1100.9	1046.9	885.0	856.1	626.6	537.9	433.8	337.4	273.8	827.1	–	0.9764	0.9743	0.2
Peggy	1131.8	684.5	1099.0	1046.9	–	869.6	632.4	536.0	435.8	337.4	273.8	829.1	–	0.9846	0.9800	0.5
Steve	1129.8	703.8	1099.0	1045.0	–	869.6	632.4	532.2	433.8	335.5	273.8	827.1	–	0.9853	0.9809	0.4
Wanda	1129.8	709.5	1099.0	1045.0	–	873.4	626.6	532.2	433.8	337.4	273.8	827.1	–	0.9788	0.9738	0.5
SAE measurements																
Olivine‐rich physical mixtures																
Bruce	1127.9	659.4	1033.4	975.6	935.1	613.1	518.7	460.8	412.6	349.0	279.6	800.2	775.1	1.0000	0.9450	5.5
Bucky	1127.9	659.4	1033.4	975.6	931.3	615.1	518.7	460.8	410.7	349.0	279.6	798.2	777.0	1.0000	0.9476	5.2
Natasha	1129.8	657.5	1033.4	975.6	931.3	613.1	518.7	462.8	410.7	350.9	279.6	798.2	775.1	0.9948	0.9425	5.2
Selina	1129.8	659.4	1031.5	975.6	927.4	613.1	518.7	460.8	410.7	350.9	281.5	800.2	775.1	0.9960	0.9473	4.9
Tony	1126.0	657.5	1033.4	975.6	935.1	615.1	518.7	460.8	410.7	349.0	279.6	798.2	775.1	1.0000	0.9512	4.9
Phyllosilicate‐rich physical mixtures																
Clint	1228.2	715.3	1083.6	1045.0	–	–	624.7	534.1	435.8	349.0	281.5	–	–	1.0000	0.9627	3.7
Nick	1237.8	713.4	1079.7	1043.1	–	–	622.8	536.0	433.8	349.0	281.5	–	–	1.0000	0.9555	4.4
Peggy	1241.7	707.6	1081.6	1043.1	–	–	628.6	534.1	435.8	347.1	279.6	–	–	1.0000	0.9465	5.3
Steve	1237.8	701.8	1083.6	1043.1	–	–	613.1	534.1	435.8	339.4	277.7	–	–	1.0000	0.9565	4.3
Wanda	1232.0	707.6	1081.6	1043.1	–	–	622.8	532.2	433.8	349.0	279.6	–	–	1.0000	0.9530	4.7

Abbreviations: CF, Christiansen feature; RB, reststrahlen bands; SAE, simulated asteroid environment; TF, transparency feature.

**Table 4:** Features (in cm^−1^) Identified in Chondritic Meteorite Spectra

Name (type)	CF1	CF2	RB1	RB2	RB3	RB4	RB5	RB6	RB7	TF 1	CF emiss	RB1 emiss	Contrast
Ambient measurements													
Orgueil (CI)	1100.9	534.1	1018.0	896.6	441.5	337.4	–	–	–	–	0.9945	0.9839	1.1
ALH 83100 (CM1/2)	1099.0	543.7	881.1	430.0	316.2	–	–	–	–	–	0.9944	0.9767	1.8
Murchison (CM2)	1045.0	620.9	877.3	505.2	416.5	323.0	–	–	–	819.4	0.9904	0.9774	1.3
Allende (CV3OxA)	1081.6	605.4	1041.1	913.9	879.2	516.7	480.1	397.2	322.0	792.4	0.9781	0.9730	0.5
Vigarano (CV3red)	1147.2	647.8	1116.3	1014.2	927.4	871.5	514.8	416.5	337.4	802.1	0.9794	0.9781	0.1
MIL 090001 (CR2)	1143.3	653.6	1072.0	1006.4	923.5	871.5	476.3	414.6	337.4	809.8	0.9804	0.9791	0.1
Farminton (L5)	1135.6	645.9	1046.9	931.3	881.1	501.3	404.9	337.4	–	804.0	0.9804	0.9680	1.2
SAE measurements													
Orgueil (CI)	1139.5	537.9	1010.3	908.1	439.6	341.3	–	–	–	–	0.9955	0.9786	1.7
ALH 83100 (CM1/2)	1089.3	545.7	877.3	430.0	314.3	–	–	–	–	–	1.0000	0.9799	2.0
Murchison (CM2)	1052.7	622.8	873.4	505.2	416.5	331.7	–	–	–	813.6	1.0000	0.9814	1.9
Allende (CV3OxA)	1083.6	597.7	1045.0	913.9	877.3	518.7	480.1	403.0	320.1	790.5	0.9887	0.9843	0.4
Vigarano (CV3red)	1147.2	651.7	1112.5	1004.5	923.5	871.5	514.8	414.6	335.5	804.0	0.9993	0.9959	0.3
MIL 090001 (CR2)	1158.8	659.4	1064.3	1002.6	923.5	871.5	474.3	416.5	335.5	823.3	1.0000	0.9840	1.6
Farminton (L5)	1164.5	651.7	1045.0	925.5	881.1	509.0	406.8	329.7	–	807.9	1.0000	0.9761	2.4

Abbreviations: CF, Christiansen feature; RB, reststrahlen bands; SAE, simulated asteroid environment; TF, transparency feature.

### 4.1 Terrestrial and Extra‐Terrestrial Minerals

Ambient and SAE spectra of the fine particulate anhydrous and hydrated silicate minerals (olivine, augite, orthopyroxene, saponite, and cronstedtite) are presented in Figure 10 and the position of spectral features and changes in spectral contrast are presented in Table 2. Ambient silicate spectra are consistent with ambient spectral measurements of similar silicate minerals (e.g., Christensen et al., 2000; Donaldson Hanna et al., 2017, 2012; Glotch et al., 2007; Hamilton, 2000, 2010; Lane & Bishop, 2019; Shirley & Glotch, 2019). For all SAE spectra of the silicate minerals, we observe a shift in the CF position to higher wavenumbers (shorter wavelengths) relative to the ambient spectra. The CFs in the saponite and orthopyroxene SAE spectra have the greatest shift (44 and 39 cm^−1^, respectively) whereas cronstedtite has the smallest shift (8 cm^−1^). We also observe a shift to lower wavenumbers (longer wavelengths) of the first RB in each SAE spectrum. However, we do not observe significant changes in position for the other identified RB. Last, we observe an increase in spectral contrast between the CF and RB in all of the silicate mineral spectra ranging from ∼2% to 13%.

Ambient and SAE spectra of fine particulate calcite are presented in Figure 10. Carbonates like calcite have RB near 1,523, 883, and 712 cm^−1^ owing to vibrations of the C‐O bonds and these bands shift to higher and lower frequencies to uniquely distinguish their mineral chemistry (Lane & Christensen, 1997, 1998). Similar to the observations of Lane and Christensen (1998) and Lane (1999), we observe an emissivity minimum near 1,544 cm^−1^ and emissivity maxima (rather than minima) near 873 and 811 cm^−1^ due to the fine particulate nature of the samples. Similar to the silicate spectra, we observe the CF position in the ambient calcite spectrum shift to higher wavenumbers in the SAE spectrum relative to the ambient spectrum and the spectral contrast increases between the CF and RB in the SAE spectrum (Table 2). The changes due to SAE conditions (CF shifts 33 cm^−1^ and the contrast increases by 8%) are on the order of changes observed in the augite and orthopyroxene SAE spectra.

Oxide minerals like magnetite and spinel have fundamental vibration bands between 800 and 200 cm^−1^ (e.g., Farmer, 1974; Christensen et al., 2000; Lane et al., 2002) and are observed in Figure 11. The ambient spectrum of fine particulate magnetite has fundamental vibration bands (RB) near 557 and 343 cm^−1^, which do not shift in position under SAE conditions. The ambient spectrum is consistent with previous measurements of magnetite (e.g., Christensen et al., 2000; Lane et al., 2002). The only difference observed between ambient and SAE conditions is a decreasing slope across the spectral range, which is related to choosing only a single maximum brightness temperature during the calibration process. Fine particulate spinel's ambient spectrum has a broad absorption band near 1,290 cm^−1^ and bands near 690 and 497 cm^−1^. No significant differences are observed between the ambient and SAE spectra. Across the thermal infrared spectral range, the ambient and SAE spectra of the fine particulate sulfides troilite and pyrrhotite are nearly featureless (spectral contrast ≤ 1%) as seen in Figure 12. Slopes are observed in both minerals' SAE spectra, similar to the slope observed in the SAE magnetite spectrum; these slopes are related to a single maximum brightness temperature chosen during the calibration process.

### 4.2 Physical Mixtures

**Figure 17:**
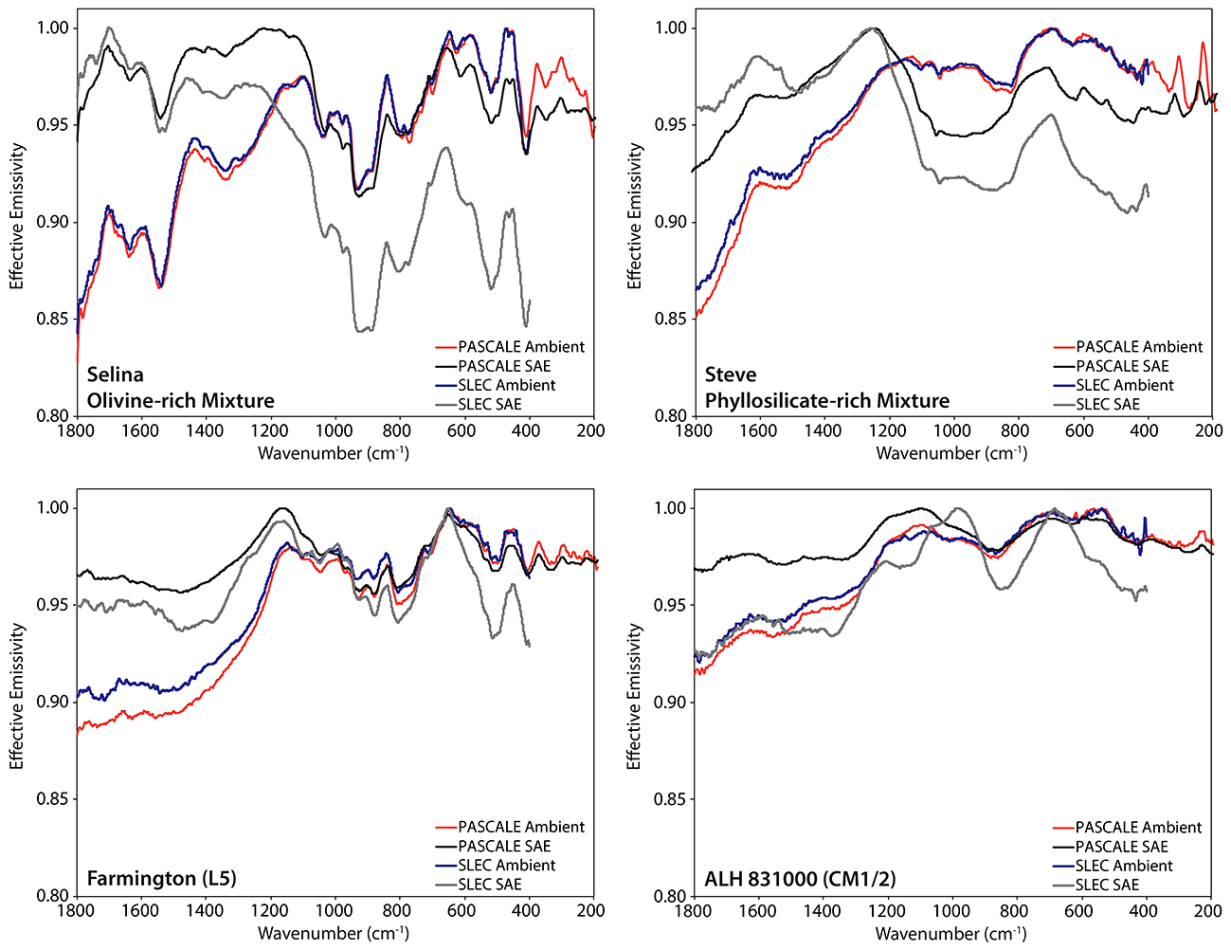
Ambient and SAE spectra of Selina (an olivine‐rich mixture), Steve (a phyllosilicate‐rich mixture), Farmington (L5), and ALH 831000 (CM1/2) as measured in PASCALE and SLEC (Donaldson Hanna et al., 2019). SLEC is attached to a FTIR spectrometer with a KBr beam splitter, which limits its spectral range to 400 cm^‐1^. FTIR, Fourier Transform Infrared; PASCALE, Planetary Analogue Surface Chamber for Asteroid and Lunar Environments; SAE, simulated asteroid environment; SLEC, Simulated Lunar Environment Chamber.

The spectral features identified in the olivine‐ and phyllosilicate‐rich physical mixtures (Figures 13 and 14 and Table 3) are consistent with those identified in the same physical mixtures measured by Donaldson Hanna et al. (2019). In Figure 17, we show ambient and SAE spectra of Selina (an olivine‐rich mixture) and Steve (a phyllosilicate‐rich mixture) as measured in PASCALE and SLEC (Donaldson Hanna et al., 2019) to demonstrate the similarities between the spectra, particularly the ambient spectra. For all mixtures measured under SAE conditions, the CF is observed to shift to higher frequencies and the spectral contrast between the CF and the RB is observed to increase relative to the ambient spectra. On average, the CF shifts to higher frequencies by ∼20 cm^−1^ for the olivine‐rich mixtures and by ∼100 cm^−1^ for the phyllosilicate‐rich mixtures and the spectral contrast increases by ∼1.5% and 4.0% for the olivine‐ and phyllosilicate‐rich mixtures, respectively. The shifts in CF position are consistent with the terrestrial and extra‐terrestrial minerals in Section 4.1 and are consistent with spectral measurements of terrestrial minerals measured under simulated lunar environment conditions (Arnold et al., 2016; Donaldson Hanna et al., 2017, 2014; Shirley & Glotch, 2019). However, the increase in spectral contrast under SAE conditions is greatly reduced in the physical mixture spectra and is related to the addition of magnetite and insoluble organic matter (IOM; low albedo materials) to the mixtures. We also observe other changes in spectral features measured in SAE conditions including (1) the secondary CF (Hamilton, 2000) shifts to higher frequencies by ∼12 cm^−1^ in the olivine‐rich mixture spectra and by ∼5 cm^−1^ in the phyllosilicate‐rich mixture spectra, (2) the RBs identified just longward (lower wavenumbers) of primary and secondary CFs shift to lower frequencies in both sets of physical mixtures, and (3) the TFs in the phyllosilicate‐rich mixture spectra are weakened to the point of disappearance. This behavior of the TFs in simulated airless body conditions was previously observed in other silicate minerals (Donaldson Hanna et al., 2012; Shirley & Glotch, 2019).

### 4.3 Chondritic Meteorites

The spectral features identified in the chondritic meteorites ambient spectra (Figures 15 and 16 and Table 4) are consistent with those identified in ambient spectra of the same meteorites measured by Donaldson Hanna et al. (2019). In Figures 8 and 17, we show ambient spectra of Murchison, Farmington, and ALH 831000 as measured in PASCALE and SLEC (Donaldson Hanna et al., 2019) to demonstrate the similarities between the spectra. The spectral behavior under simulated asteroid conditions is highly dependent on the composition of the meteorite. The SAE spectra of Farmington (L5) and MIL 090001 (CR2) show shifts in the CF to higher frequencies by 29 and 15 cm^−1^ and spectral contrast increases of 1% and 2%, respectively. These shifts in CF position are similar to those observed in the SAE spectra of the olivine‐bearing physical mixtures, which is consistent with Farmington and MIL 090001 having a significant amount of olivine in their composition (Donaldson Hanna et al., 2019). The SAE spectra of the CV and CM carbonaceous chondrites (Allende, Vigarano, Murchison, and ALH 83100) show little to no shift in the CF position and spectral contrast increases on the order of 0%–1%. The SAE spectrum of Orgueil (CI) shows the largest shift in CF position (39 cm^−1^) for all of the meteorites. The spectral contrast increases in all the SAE spectra of the chondritic meteorites (average of 1%) are lower than that of the physical mixtures (average of 3%) and minerals (average of 7%), which results from a reduced thermal gradient in the upper hundreds of microns of the sample due to the low albedo nature of the meteorites (Donaldson Hanna et al., 2019; Salisbury et al., 1991).

## 5 Discussion

Our ambient and SAE spectral measurements of the fine particulate (<90 and < 45 μm) terrestrial and extra‐terrestrial silicate minerals are consistent with recent laboratory spectra of lunar and asteroid analogs measured under ambient and simulated lunar environment (SLE) conditions (Arnold et al., 2016; Donaldson Hanna et al., 2017, 2014; Shirley & Glotch, 2019). We observe changes in the emissivity spectra due to the changes in the environmental conditions. Specifically, the CF shifts to higher frequencies (shorter wavelengths) and the spectral contrast between the CF and the RB increases under SAE conditions. These changes are expected as they have previously been observed (e.g., Arnold et al., 2016; Donaldson Hanna et al., 2014, 2017, 2012; Henderson et al., 1996; Logan & Hunt, 1970; Logan et al., 1973; Salisbury & Walter, 1989; Shirley & Glotch, 2019). Mg‐rich (forseritic) olivine samples have been investigated here and in other previous studies, which allows for a comparison between spectral measurements. The primary and secondary CF positions we identified in the San Carlos olivine (Fo_∼90_, where Fo# represents the fraction of Mg to Mg + Fe) ambient and SAE spectra are at similar frequencies to published values for other samples having similar Fo# (Arnold et al., 2016; Donaldson Hanna et al., 2017, 2012; Hamilton, 2010; Shirley & Glotch, 2019). In addition, the observed increase in spectral contrast between ambient and SAE conditions is similar to spectral contrast increases between ambient and SLE conditions (Donaldson Hanna et al., 2017; Shirley & Glotch, 2019). Thus, our terrestrial and extra‐terrestrial mineral spectra are comparable with emissivity spectra of mineral end members from other laboratories.

Spectra of the olivine‐bearing physical mixtures can be compared to spectra of San Carlos olivine to understand the effects of albedo as the mixtures contain up to 5 wt.% magnetite, a low albedo material (Donaldson Hanna et al., 2019). The olivine‐bearing mixtures were designed to have mineral abundances similar to type‐3 carbonaceous chondrite meteorites whose mineralogy is dominated by olivine like Allende. Allende, a CV3_OxA_ meteorite, has an average albedo of ∼0.085 or 8.5% (Beck et al., 2012). Thus, to further understand the effects of albedo, we can also compare the spectral contrast of features in the pure olivine and mixture spectra with the spectral contrast of features in the Allende spectrum. For the ambient spectra, the spectral contrast between the CF and the first RB of the olivine‐bearing mixtures range from 3.1% to 4.2% whereas the spectral contrast for San Carlos olivine is 13.4%. For the SAE spectra, the spectral contrast between the CF and the first RB of the olivine‐bearing mixtures range from 4.9% to 5.5% whereas the spectral contrast for San Carlos olivine is 19.9%. The spectral contrast between the CF and the first RB of the ambient Allende spectrum is 0.5% and 0.4% in the SAE spectrum. In addition, the greatest spectral contrast observed across the entire spectral range is the emissivity difference between the TF and the secondary CF, which is 4.8% in the ambient spectrum and 4.0% in the SAE spectrum. Two observations can be made: (1) the decrease in albedo results in a reduction in the spectral contrast of features across the entire spectral range and (2) the decrease in albedo reduces the thermal gradient under SAE conditions, which reduces the change in spectral contrast between ambient and SAE conditions. In addition, we also observe little to no shift in the CF position in Allende's spectrum between ambient and SAE conditions, which is also related to the reduced thermal gradient. Similar observations were made by Shirley et al. (2017) when olivine was darkened by incrementally adding carbon black to a fine particulate sample.

We also compared the PASCALE ambient and SAE spectra of the physical mixtures and chondritic meteorites to the SLEC ambient and SAE measurements of the same samples (Donaldson Hanna et al., 2019). Figure 17 includes ambient and SAE spectra of Selina (olivine‐rich mixture), Steve (phyllosilicate‐rich mixture), Farmington, and ALH 831000 and Figure 8 includes ambient spectra of Murchison. Ambient spectra from the two chambers are consistent with each other and spectral features identified in the ambient SLEC spectrum are identified at the same wavenumber position in the PASCALE spectrum. We do observe subtle differences in the slopes of the spectra, which are likely due to differences in the calibration of the experimental two setups, particularly the effects of downwelling radiance. Additionally, we observe differences between the SAE spectral measurements for the CM (Murchison and ALH 83100) and CV (Allende and Vigarano) meteorites. The SLEC spectra of the CM and CV meteorites show an increase in the spectral contrast near 1,000 cm^−1^ and between the TF and secondary CF (Donaldson Hanna et al., 2019). These enhancements in spectral contrast are not observed in the PASCALE measurements and we must consider what is causing these differences.

We have demonstrated the radiometric stability of PASCALE (Figures 4, 5, and 17) and spectral comparisons of mineral end members and physical mixtures with other measurements has demonstrated the reliability of the system in producing calibrated spectra that are similar to those in the literature (Figures 9, 10, 11, 12, 13, 14, 15, 16). We have also shown that the methods used for preparing sample materials produce repeatable results (Figure 8). Thus, we believe the methods we used to collect and calibrate the PASCALE measurements are sound. We must then consider the possible differences between the PASCALE and SLEC systems to understand these observed SAE spectral differences. First, we consider the differences in the evacuation rates of the turbomolecular vacuum pumps between the two environment chambers. It is possible that the vacuum pump on SLEC has altered the near‐surface of the CM and CV meteorite samples and increased their porosities. However, it is not clear why the CM and CV meteorite samples would be preferentially altered and no obvious changes to the samples were observed when the samples were removed from the chambers. Next, we consider the differences in the incidence angle of the quartz halogen lamp onto the sample between the two chambers. SLEC's lamp has a 30° incidence angle while PASCALE's lamp has a 55° incidence angle. Recent work by Warren et al. (2019) has shown that the emissivity of a randomly rough surface has an angular dependence, which suggests that the observed SAE spectral differences might be related to the different lamp geometries. Since the SLEC and PASCALE measurements are a first of their kind, further SAE measurements of the CV and CM meteorites are needed to better understand the observed contrast enhancements in the SLEC spectra.

## 6 Conclusions

We demonstrate the capabilities of a bespoke vacuum environment chamber (PASCALE) in simulating the near‐surface conditions of airless bodies. Repeat measurements of the internal calibration target over a three‐month period were used to highlight the radiometric stability of the combined system (PASCALE and the Bruker FTIR spectrometer) and showed that the instrument response function was stable to ≤0.25% under ambient and simulated asteroid environment conditions. We made repeat measurements of the Murchison meteorite sample to demonstrate the reliability of our sample preparation technique, which showed that the differences in repeat ambient measurements are smaller than the uncertainties in an ambient emissivity measurement.

We measured a suite of samples previously characterized under ambient and simulated asteroid environment conditions using a different experimental set‐up. This sample suite included two sets of physical mixtures, one dominated by olivine and the other by phyllosilicates, and chondritic meteorites. In addition, for the first time, we measured the terrestrial and extra‐terrestrial minerals that were used to make the physical mixtures. Our lab measurements show that the CFs identified in the physical mixture SAE spectra shift to higher frequencies in a similar fashion to the CFs identified in mineral SAE spectra. However, the addition of low albedo materials (e.g., magnetite and insoluble organic material) to the physical mixtures reduces the amount of spectral contrast change under SAE conditions. SAE spectra of the anhydrous meteorites (Farmington, Allende, Vigarano, and MIL 090001) have similar spectral behavior as the olivine‐bearing physical mixtures, but the spectral contrast is further reduced due to the low albedo nature of the meteorite samples. Our SAE spectra of the hydrated meteorites (Orgueil, Murchison, and ALH 83100) show little to no difference when compared to their ambient spectra suggesting the low albedo nature of hydrated carbonaceous chondrites reduces the thermal gradient to the point it has negligible effects on the thermal infrared signature. Thus, the thermal infrared signature measured under airless body conditions is highly dependent on the composition and albedo of the surface material.

## Supporting information

Supporting Information S1

## Data Availability

All calibrated emissivity spectral data can be found on the University of Oxford's Research Archive (Bowles & Donaldson Hanna, 2020).
