# Supplementary material for: Spectral Characterization of Bennu Analogs Using PASCALE: A New Experimental Set‐Up for Simulating the Near‐Surface Conditions of Airless Bodies
Source: J Geophys Res Planets. 2021 Feb 18;126(2):e2020JE006624. doi: 10.1029/2020JE006624 (PMC7988566; doi:10.1029/2020JE006624)
Supplement: Supplementary file 1 — Supporting Information S1 [file JGRE-126-e2020JE006624-s001.docx]

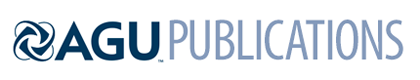


*Journal of Geophysical Research Planets*

Supporting Information for

**Spectral Characterization of Bennu Analogs Using PASCALE: A New Experimental Set-up for Simulating the Near-Surface Conditions of Airless Bodies**

K. L. Donaldson Hanna^1,2^, N. E. Bowles^2^, T. J. Warren^2^, V. E. Hamilton^3^, D. L. Schrader^4^, T. J. McCoy^5^, J. Temple^2^, A. Clack^2^, S. Calcutt^2^, and D. S. Lauretta^6^

^1^Department of Physics, University of Central Florida, Orlando, FL, USA, ^2^Atmospheric, Oceanic, and Planetary Physics, University of Oxford, Oxford, UK, ^3^Department of Space Science, Southwest Research Institute, Boulder, CO, USA, ^4^Center for Meteorite Studies, Arizona State University, Tempe, AZ, USA, ^5^Smithsonian National Museum of Natural History, Washington D. C., USA, and ^6^Lunar and Planetary Laboratory, University of Arizona, Tucson, AZ, USA

**Contents of this file**

Text S1

References

**Introduction**

This file describes the calibrated emissivity spectral data found on the University of Oxford’s Research Archive (Bowles and Donaldson Hanna, 2020). The calibrated spectral data are provided as ASCII text files. Each text file includes a five-line header followed by two columns of data. The header includes the filename, the Oxford calibrated data file name, the environmental conditions under which the sample was measured, the version of the calibration code used in processing the data file, the spectral range over which the maximum brightness temperature was searched for, and the column headers. The first column of data is wavenumber (cm^-1^) and the second is effective emissivity, which is an averaged value of three measurements.

Text S1.

Below are example file headers. The first is an example for an ambient spectrum and the second is an example for a simulated asteroid environment (SAE) spectrum. Ambient spectra are identified in two ways: (1) the filename ends *_001_E.txt and (2) the third line in the header includes Nitrogen80c. SAE spectra are identified by: (1) the filename ends *_002_E.txt and (2) the third line in the header includes SAE60c. Thus, each sample measured for the manuscript will have two files in the archive.

OREX_BRUCE_001_E.txt

Oxford calibrated filename= 20180316134003_KDH_OREx_Bruce_80C_N2_emissivity.csv

Nitrogen80c BB calibration emissivity_calc_average_J_BB and CF calibration 1700 to 400 cm-1

Wavenumber (cm-1)

Emissivity

OREX_BRUCE_002_E.txt

Oxford calibrated filename= 20180403154341_KDH_Bruce_60C_Lamp_9p2V_emissivity.csv

SAE60c BB calibration emissivity_calc_average_J_BB and CF calibration 1700 to 400 cm-1

Wavenumber (cm-1)

Emissivity

Analog samples measured in this investigation include minerals, anhydrous and hydrated physical mixtures, and meteorites. Mineral filenames include the locality from which the sample was collected (if it is known) and the particle size distribution of the sample.

Minerals:

CRONSTEDTITE_LT90_00*_E.txt

JOHNSTOWN-ORTHOPYROXENE_LT90_00*_E.txt

KAKANUI-AUGITE_LT90_00*_E.txt

MINASGERAIS-MAGNETITE_LT90_00*_E.txt

MONOLAKE-SAPONITE_LT90_00*_E.txt

MUNDRABILLA-TROILITE_LT90_00*_E.txt

NEWMEXICO-CALCITE_LT90_00*_E.txt

PYRRHOTITE_LT90_00*_E.txt

SANCARLOS-OLIVINE_LT90_00*_E.txt

SPINEL_LT45_00*_E.txt

The physical mixture filenames include the name of the physical mixtures from Donaldson Hanna et al. (2019). Since the physical mixtures were created as part of a blind test, the filenames are not representative of minerals included in the mixtures.

Anhydrous Physical Mixtures (olivine-rich mixtures):

BRUCE_00*_E.txt

TONY_00*_E.txt

NATASHA_00*_E.txt

BUCKY_00*_E.txt

SELINA_00*_E.txt

Hydrated Physical Mixtures (phyllosilicate-rich mixtures):

STEVE_00*_E.txt

CLINT_00*_E.txt

PEGGY_00*_E.txt

WANDA_00*_E.txt

NICK_00*_E.txt

The chondritic meteorite filenames include the name of the meteorites from Donaldson Hanna et al. (2019). Since the chondritic meteorites were created as part of a blind test, the filenames are not representative of the actual meteorite. Below are the names of each meteorite and its corresponding filename.

Meteorites:

Allende SHEPARD_00*_E.txt

Farmington GRISSOM_00*_E.txt

Murchison GLENN_00*_E.txt

MIL 090001 CARPENTER_00*_E.txt

ALH 83100 SCHIRRA_00*_E.txt

Orgueil COOPER_00*_E.txt

Vigarano SLAYTON_00*_E.txt

References.

Bowles, N., and Donaldson Hanna K. (2020), PASCALE Spectral Data for OSIRIS-REx [Data set], University of Oxford, https://doi.org/10.5287/BODLEIAN:4R5RW0DQG.

Donaldson Hanna, K. L., Schrader, D. L., Cloutis, E. A., Cody, G. D., King, A. J., McCoy, T. J., Applin, D. M., Mann, J. P., Bowles, N. E., Brucato, J. R., Connolly Jr., H. C., Dotto, E., Keller, L. P., Lim, L. F., Clark, B. E., Hamilton, V. E., Lantz, C., Lauretta, D. S., Russell, S. S., and Schofield, P. F. (2019), Spectral characterization of analog samples in anticipation of OSIRIS-REx’s arrival at Bennu: A blind test study. *Icarus,* 319, 701-723. doi:10.1016/j.icarus.2018.10.018.
